# Discovering a predictive metabolic signature of drug-induced structural cardiotoxicity in cardiac microtissues

**DOI:** 10.1007/s00204-025-04074-4

**Published:** 2025-05-16

**Authors:** Tara J. Bowen, Andrew R. Hall, Gavin R. Lloyd, Matthew J. Smith, Ralf J. M. Weber, Amanda Wilson, Amy Pointon, Mark R. Viant

**Affiliations:** 1 School of Biosciences, University of Birmingham, Edgbaston, Birmingham, B15 2 TT UK; 2 Safety Sciences, Clinical Pharmacology and Safety Sciences, BioPharmaceuticals R&D, AstraZeneca, Cambridge, UK; 3 Phenome Centre Birmingham, University of Birmingham, Edgbaston, Birmingham, B15 2 TT UK; 4 Integrated Bioanalysis, Clinical Pharmacology and Safety Sciences, BioPharmaceuticals R&D, AstraZeneca, Cambridge, UK; 5 Present Address: Medicines Discovery Catapult, Alderley Park, Cheshire, SK10 4 TG UK

**Keywords:** Structural cardiotoxicity, Metabolic biomarkers, Hybrid metabolomics, 3D triculture cardiac model, ADME/TK, MTox700+

## Abstract

Improved prediction of drug-induced structural cardiotoxicity is required to reduce attrition driven by cardiac safety concerns in drug discovery. Omics measurements are well suited to this need, offering the potential to discover molecular signatures associated with toxicological endpoints. In addition, untargeted metabolomics can simultaneously measure xenobiotic fate within the test system. We present an extensive metabolomics study to discover a predictive metabolic signature of drug-induced structural cardiotoxicity. A human-relevant in vitro cardiac model, cardiac microtissues, were exposed to twelve xenobiotics (eight clinically labelled structural cardiotoxins and four non-cardiotoxic pharmaceuticals), each at two concentrations, for 6, 24, and 48 h. The measurements were made by direct-infusion and liquid-chromatography mass spectrometry from intracellular polar and lipid extracts, and spent culture medium, respectively. Data were used to quantify levels, and reveal the metabolic fate of the xenobiotics, and to simultaneously explore their effects on the cardiac microtissues. Xenobiotic quantification revealed free concentrations to be typically lower than nominal values, whilst discovery of xenobiotic-related features evidenced the biotransformation capacity of the microtissues. Both common and condition-specific effects of the xenobiotics on the intracellular metabolome, lipidome, and metabolic footprint were discovered. Moreover, metabolic signatures with capacity to predict structural cardiotoxicity were revealed. These included features representing several ceramides, energy metabolism intermediates, e.g. creatine, purine-related metabolites, and markers of oxidative stress, e.g. glutathione.

**Supplementary Information:**

The online version contains supplementary material available at 10.1007/s00204-025-04074-4.

## Introduction

Drug-induced structural cardiotoxicity (SCT) poses a significant challenge in the development of safe and efficacious medicines, evidenced by the large proportion of market withdrawals and project closures driven by cardiac safety concerns (Cook et al. 2014; Onakpoya et al. 2016). The inability to accurately predict cardiac safety liabilities, particularly those causing morphological damage to cardiac tissue, (i.e. SCT), during the earlier phases of drug development has been linked to costly late-stage failures (Laverty et al. 2011). Recent attempts to address this challenge have focussed on developing in vitro assays targeting established organelle-level pathologies associated with SCT, e.g. high content biology (HCB) to measure the effect on mitochondrial membrane permeability (Δψm), and endoplasmic reticulum (ER) (Archer et al. 2018), and other proposed key events, e.g. panel of biomarkers against apoptosis, oxidative stress, lipid accumulation and myocardial injury (Doherty et al. 2013, 2015). Notable is the use of human induced pluripotent stem cell-derived cardiomyocytes (hiPSC-CMs) in 3D co-cultures with other major cardiac cell types, mainly endothelial cells and fibroblasts, i.e. cardiac microtissues, as the in vitro test system to maximise translational relevance and improve the sensitivity of the assays (Pointon et al. 2017; Ravenscroft et al. 2016a, b; Ravenscroft et al. 2016b; Giacomelli et al. 2017; Archer et al. 2018). Whilst these in vitro assays are promising steps towards improved prediction of SCT, further efforts are required to discover translational predictive biomarkers.

Omics approaches are at the forefront of investigative toxicology, driven by their ability to measure molecular perturbations that can be associated with a mode-of-action (MoA) or that can lead to discovering predictive biomarkers (Alexander-Dann et al. 2018; Campos and Colbourne 2018; Brockmeier et al. 2017; Nguyen et al. 2022). Untargeted metabolomics measures the collection of small molecules representing the substrates, intermediates, and end products of a system’s metabolism (Ramirez et al. 2013; Alonso et al. 2015; Viant et al. 2019; Alarcon-Barrera et al. 2022). Previous untargeted metabolomics investigations of SCT have included profiling doxorubicin-induced metabolic changes in the heart and plasma of B6 C3 F1 mice (Schnackenberg et al. 2016), in plasma of rats (Wang et al. 2009), and in the footprint of hiPSC-CMs (Chaudhari et al. 2017). Perturbation of the rat plasma metabolome induced by cyclophosphamide (Yin et al. 2016) and doxorubicin, isoproterenol and 5-fluorouracil (Li et al. 2015), and of the plasma, heart, muscle and liver metabolomes of rats following sunitinib and sorafenib treatment (Jensen et al. 2017a, b; Jensen et al. 2017b), have also been explored. However, these studies have been limited to investigating just one or a few xenobiotics at a time. The specificity and generalisability of any proposed biomarkers or mechanisms have not been evaluated with respect to a broad range of known structural cardiotoxins, limiting their application in drug discovery-based screening. More recently, Palmer et al. (2020) conducted an extensive study, profiling the metabolic responses of hiPSC-CMs to 66 xenobiotics, including structural and functional cardiotoxins and non-cardiotoxic xenobiotics. These data were used to develop a four-metabolite-based targeted assay predictive of cardiotoxicity potential, confirmed by test data on 88 xenobiotics. However, this study used a test system with restricted translational relevance—2D monolayers lack physiologically relevant cell–cell and cell–extracellular matrix (ECM) interactions, and non-myocytes, which represent 70% of the cardiac cell mass and play essential roles in modulating the structure and function of myocytes in vivo. Also, only the metabolic footprint was measured, representing a minor proportion of the total intracellular metabolic network. Addressing these limitations may enable discovery of a more comprehensive biomarker panel with improved predictivity and translational relevance.

The fate of xenobiotics in in vitro systems used during drug discovery and preclinical screening is often overlooked. However, evidence demonstrates that free concentrations often differ significantly from nominal concentrations, which can have negative implications on concentration–response modelling and in-vitro-to-in-vivo extrapolation (IVIVE) (Groothuis et al. 2015). Furthermore, the biotransformation capacity of most non-hepatic test systems remains uncharacterised and usually assumed to be negligible, despite the relevance of xenobiotic biotransformation to predicting clinical toxicity outcomes (Coecke et al. 2006, 2013). A consequence of the unbiased nature of untargeted metabolomics is the potential measurement of test xenobiotics and their biotransformation products (BTPs). As demonstrated previously, these untargeted metabolomics-based measurements can be exploited to discover the fate of exposure xenobiotics in test systems (Bowen et al. 2023; Sostare et al. 2024). Furthermore, absolute quantitation of parent xenobiotics can be incorporated into ultra-high-performance liquid chromatography coupled mass spectrometry (UHPLC-MS)-based metabolomics, enabling validation of exposure concentrations (Bowen et al. 2021; Groothuis et al. 2015). Inclusion of these capabilities into a metabolomics-based in vitro toxicology study is highly attractive towards maximising the potential translational relevance and accuracy of IVIVE.

The overall aim of this study was to apply metabolomics to measure the fate and effects of eight (clinically recognised) structurally cardiotoxic, and four non-structurally cardiotoxic, xenobiotics in cardiac microtissues, towards discovering a metabolic signature predictive of SCT. The first objective was to quantify the extracellular free concentrations of the twelve parent xenobiotics and discover their routes of biotransformation, to reveal their fate and to evaluate the biotransformation competency of cardiac microtissues. The second objective was to characterise the response of cardiac microtissues induced by two concentrations of 12 parent xenobiotics after exposure for 6, 48 and 72 h. This included measuring the effects on the intracellular polar metabolic and lipid fingerprints, and on the extracellular metabolic footprint using nano-electrospray direct infusion mass spectrometry (nESI-DIMS) and UHPLC-MS metabolomics, respectively. The exposure concentrations were selected based on previous work (Archer et al. 2018) with the aim of investigating structural cardiotoxicity-specific molecular mechanisms and not gross pathophysiology. The final objective was to discover metabolic signature(s) in the cardiac microtissues predictive of SCT through multivariate analysis of the metabolomics data.

## Materials and methods

### Cell culturing, exposures and sample collection

Cell culturing and microtissue formation were performed as described previously (Bowen et al. 2021). Test xenobiotics were selected from a list of 30 U.S. Food and Drug Administration approved and marketed small molecule drugs for which in vitro toxicity data were available in the form of HCB measurements (ER integrity and Δψm) in cardiac microtissues, measured after 72-h exposures (Archer et al. 2018, Supplementary Methods). The nominal exposure concentrations used in the metabolomics study (Table 1) were selected based on these HCB measurements.

**Table 1:** The nominal low and high concentrations for each of the 12 xenobiotics to which cardiac microtissues were exposed in this study

Batch	Xenobiotic	Structurally cardiotoxic	Nominal concentration—low (μM)	Nominal concentration—high (μM)	Reason for nominal high concentration
A	Acyclovir	False	15.8	99.9	15 × *C*_max_
A	Clozapine	True	2.23	7.05	15 × *C*_max_
A	Sorafenib	True	1.18	3.74	Cell viability IC_30_
B	Buspirone	False	0.14	0.45	15 × *C*_max_
B	Doxorubicin	True	0.11	0.36	Cell viability IC_30_
B	Lapatinib	True	1.75	5.53	Cell viability IC_30_
C	Erlotinib	False	14.2	45.0	15 × *C*_max_
C	Idarubicin	True	0.095	0.30	Lowest investigated
C	Sunitinib	True	1.12	3.53	Cell viability IC_30_
D	Mebendazole	False	9.63	30.5	15 × *C*_max_
D	Dasatinib	True	3.42	10.8	15 × *C*_max_
D	Fluorouracil	True	15.8	69.2	15 × *C*_max_

The reasoning for the selected nominal high concentration is reported, as per the following criteria: lowest value from (1) IC_30_ cell viability at 72 h after initial exposure, as indicated by results of an ATP depletion assay, i.e. a phenotypically anchored concentration, (2) 15 × *C*_max_, to ensure therapeutic relevance, and (3) the lowest nominal concentration previously investigated by HCB. The low exposure concentrations were a half-log dilution of the high exposure concentrations; these are concentrations at which no ATP depletion was observed. Also detailed is the batch in which exposure to each xenobiotic was carried out, and the SCT label for each xenobiotic

Exposures were conducted as described previously (Bowen et al. 2021) across four separate batches, using 21-day mature cardiac microtissues. Each batch included exposure to two structurally cardiotoxic xenobiotics and one non-structurally cardiotoxic xenobiotic, each at two concentrations, for 6, 48 and 72 h (Table 1). Each batch also included biological controls, where microtissues were exposed to 0.1% DMSO for 6, 48 or 72 h.

The microtissues and spent culture media were collected for metabolomics analysis as described previously (Bowen et al. 2021). Each sample of 28 microtissues was isolated on the filter surface of a cell strainer and washed with 0.9% sodium chloride and then ultrapure water. The corresponding filtered spent culture medium was collected into a microcentrifuge tube.

### Extraction of intracellular polar compounds and lipids

Extraction of intracellular polar metabolites and lipids from cardiac microtissues was performed as described previously (Bowen et al. 2021). The extraction solvents for biological samples, i.e. 4:1 methanol/water and 3:1 methanol/chloroform, were supplemented with 0.2 μM L-tryptophan-(indole-d5) (Merck, Gillingham, UK) and 0.2 μM dodecylphosphorylcholine-d38 (Merck) internal standards for polar metabolites and lipids, respectively. Extracts were transferred to 96-well microplates prior to drying. Intra-study quality control (QC) aliquots were derived from a pool of biological control extracts. All dried extracts were stored at − 80 °C until analysis.

### Data acquisition by nESI-DIMS

The polar extracts were resuspended in 30 μL 4:1 (v/v) methanol/water with 0.25% formic acid and 4:1 (v/v) methanol/25 mM aqueous ammonium acetate for positive and negative ion metabolomics, respectively. Lipid extracts were resuspended in 40 μL 2:1 (v/v) 7.5 mM methanolic ammonium acetate/chloroform (Supplementary Methods).

Data were acquired using an Orbitrap ID-X Tribrid mass spectrometer (Thermo Scientific) with a nESI source (TriVersa Nanomate, Advion) and a modification of the spectral stitching nESI-DIMS method reported previously (Southam et al. 2016; Malinowska et al. 2022, Supplementary Methods). Analysis was performed across two analytical batches. Intra-study QCs were analysed after every eighth sample. Process blank data were acquired at the start and end of each exposure batch block. MS^n^ data were acquired at the end of each analytical batch.

### Processing and annotation of nESI-DIMS data

Data were processed using the DIMSpy tools (Weber and Zhou 2020) in Galaxy interface as described previously (Southam et al. 2016; Bowen et al. 2021, Supplementary Methods). Putative xenobiotic-related features within the processed datasets were discovered and annotated using a previously developed workflow (Bowen et al. 2023, Supplementary Methods). Processed data were split into batch-specific peak-intensity matrices before further filtering and pre-processing using the R/Bioconductor package, structToolbox (Lloyd et al. 2021), to prepare the data for statistical analysis (Supplementary Methods).

Putative annotation (Metabolomics Standards Initiative, MSI, level 3, Sumner et al. 2007) of endogenous *m/z* features was achieved using the Python package BEAMSpy (v0.1.0). Ion forms were annotated against a list of common adducts, isotopes, neutral losses and multiply charged species, according to calculated mass differences, with mass tolerance of ± 2 ppm. Compound annotation was performed using the curated lists of metabolites and lipids present in cardiac microtissues (Bowen et al., 2025) and list of toxicity metabolic biomarkers (MTox700+, Sostare et al. 2022) as the reference compound library and a ± 2 ppm mass error window. More confident annotations (MSI levels 1–2, Sumner et al. 2007) were derived by spectral matching of processed MS^n^ data to in-house library of toxicologically relevant compounds and public spectral databases (MassBank of North America, http://mona.fiehnlab.ucdavis.edu) using the Python package MSnPy (https://github.com/computational-metabolomics/msnpy), operated in a Galaxy interface (Supplementary Methods).

### Data acquisition by UHPLC-MS

The samples of spent culture medium (504 samples, *N* = *6* per exposure group) were prepared as described previously (Bowen et al. 2021), in 96-well plates, with some modifications. Intra-study QCs were derived from pooled aliquots of all study samples. Additionally, 12 pools of aliquots from high concentration exposed samples, one for each test xenobiotic, and a pool of aliquots from every control sample were generated. Process blanks were generated using water, instead of media. Quantitation calibration standards, quantitation quality control standards (quant-QCs), and quantitation blank samples (quant-blanks) were prepared as described previously (Bowen et al. 2021, Supplementary Table 3). All aliquots were prepared for analysis by adding 150 μL 1:1 acetonitrile/methanol containing 1 μM L-tryptophan-(indole-d5), followed by centrifugation at 2600 g for 10 min. The supernatants were transferred to 96-well microplates for subsequent analysis.

The samples were analysed by hydrophilic-interaction liquid chromatography (HILIC)-based UHPLC-MS(/MS) as described previously (Bowen et al. 2021). Quantitation calibration standards, quant-QCs, and quant-blanks were analysed at the start and end of each exposure batch block. Intra-study QCs were analysed after every sixth sample and process blanks were acquired at the start, middle and end of the analytical sequence. Xenobiotic-related MS/MS data were acquired at the end of each exposure batch block. Endogenous-related MS/MS data were acquired twice, in the middle and at the end of the analytical run.

### Quantitation of extracellular parent xenobiotic concentrations

Data processing for xenobiotic quantitation was performed using TraceFinder (v5.1, Thermo Scientific). Calibration functions were calculated by least-squares non-linear regression analysis with a weighting factor of 1/*x*^2^. The goodness-of-fit was evaluated using the *R*^2^ value.^.^ Calibration standard measurements were excluded from the final regression analysis if the absolute difference between the back-calculated and nominal concentrations exceeded 40%. The accuracy and precision of calibration was assessed using accepted quant-QCs (with an absolute difference between back-calculated and nominal concentrations ± 40%). Accuracy was defined as the mean calculated concentration relative to nominal concentrations, and precision was determined as the mean relative standard deviation (RSD) across the calculated concentrations of duplicate quant-QCs.

### Processing and annotation of UHPLC-MS untargeted data

Raw UHPLC-MS untargeted metabolomics data were processed as described previously using XCMS (v3.12.0) after conversion to mzML file format using ProteoWizard software (v3.0.19198) (Bowen et al. 2021, Supplementary Methods). Xenobiotic-related features within the dataset were discovered and annotated using the method reported previously (Bowen et al. 2023, Supplementary Methods).

Endogenous peak matrix processing was performed as described previously, per batch (Bowen et al. 2021, Supplementary Methods). Quality control-based robust spline correction (QC-RSC) (Kirwan et al. 2013) was applied to correct for drift in intensity as a function of injection order, after removal of blank and xenobiotic-related features, and prior to other filtering.

Putative metabolite annotation (MSI level 3, Sumner et al. 2007) was achieved using the Python package BEAMSpy (v0.1.0). Features were grouped according to retention time (RT) (± 5-s tolerance) and Pearson’s correlation coefficient (*r* ≥ 0.7) and *p*-value (*p* ≤ 0.01). Ion forms were annotated against a list of common adducts, isotopes, neutral losses and multiply charged species, according to calculated mass differences, with mass tolerance of ± 5 ppm. Compound annotation was performed using Human Metabolome Database (HMDB, v4.0, accessed 9 th September 2020) as the reference compound library and a ± 5 ppm mass error window. More confident annotations (MSI levels 1–2, Sumner et al. 2007) were derived by RT and spectral matching to an in-house RT library and spectral database, respectively, of toxicologically relevant metabolites, i.e. metabolites within MTox700 + list of biomarkers (Sostare et al. 2022), and by spectral matching against mzCloud database, performed using Compound Discoverer (v3.3, Thermo Scientific, Supplementary Methods).

### Statistical analysis

Univariate analyses (Welch’s *t-*test and fold change calculations) were performed on normalised peak-intensity matrices using the R/Bioconductor packages structToolbox (v1.6.1, Lloyd et al. 2021). Intensity measurements in samples corresponding to each exposure condition (exposure xenobiotic, concentration, and duration) were compared to batch- and exposure duration-matched controls (Supplementary Methods).

Orthogonal partial least squares discriminatory analysis (OPLS-DA) was performed using the R/Bioconductor packages struct and structToolbox (Lloyd et al. 2021) on matrices of control normalised responses, i.e. relative intensities in xenobiotic exposed samples were normalised against median intensity in batch- and exposure duration-matched controls, to correct for any batch- and baseline temporal-based variation (Supplementary Methods). Prior to analysis, data were log_2_ transformed, missing values imputed by *k*-nearest neighbour (KNN, *k* = 5), and vector normalised.

## Results and discussion

### Discovering the fate of xenobiotics in a cardiac microtissue test system

### Quantifying the free concentration of parent xenobiotics

**Fig. 1:**
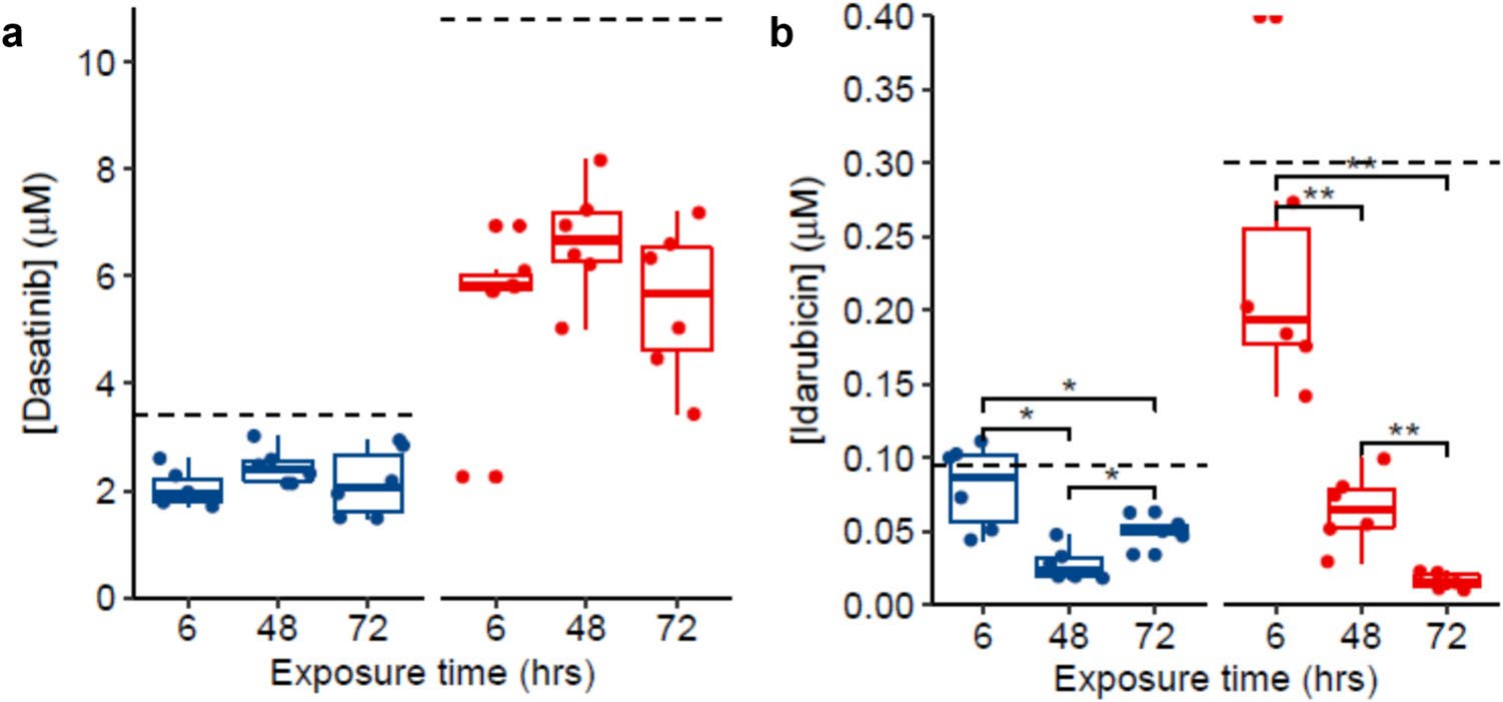
Quantitation of parent xenobiotics in spent culture medium of cardiac microtissues. Representative boxplots displaying the concentrations of **a** dasatinib, and **b** idarubicin samples of pooled spent culture medium from 28 cardiac microtissues, incubated for 6, 48 and 72 h (hrs) following addition of the xenobiotics at the nominal concentrations. Concentrations were calculated by external calibration. Blue and red represent calculated concentrations in samples spiked with nominal low and high concentration, respectively. The dashed lines represent the nominal concentrations. Individual points show data from *N* = 6 replicates. * and ** represent *p* < 0.05 and *p* < 0.01 significant difference between measured concentrations at each time point (two-sided t-test with Holm’s multiple test correction) (Color figure online)

In this study, cardiac microtissues were exposed to one of twelve small molecule xenobiotics for 6, 48 or 72 h at two nominal concentrations (‘low’ and ‘high’; Table 1). Targeted absolute quantitation of these xenobiotics, using the same UHPLC-MS analysis that measured the endogenous metabolites, enabled comparison of nominal exposure concentrations to the free (i.e. fully dissolved and not bound to medium constituents or adsorbed to plasticware) concentrations to which microtissues were exposed, over the duration of the experiment (Fig. 1, Supplementary Figs. 6–9, Supplementary Table 4). Eleven of twelve xenobiotics were reliably quantified, whilst concentrations of doxorubicin could not be determined since either the corresponding chromatographic peak was not detected, intensities were below the threshold for quantitation, or calculated concentrations were less than the lower limit of quantitation. Concentrations of the eleven quantifiable xenobiotics in stability control samples (nominal high concentration of each xenobiotic incubated in culture medium only for 6, 48 and 72 h) were also determined, to elucidate the chemical stability under the experimental conditions (Supplementary Fig. 10).

The free concentrations of all 11 quantifiable xenobiotics were significantly lower than nominal concentrations at one or more exposure levels (low or high) in both biological and stability control samples, when measurements from all exposure durations were combined (Fig. 1a, Supplementary Figs. 9 and 10, Supplementary Table 5). These lower than nominal concentrations may be attributed to non-specific binding to culture medium constituents such as serum, or adsorption to the plastic culture vessel (Groothuis et al. 2015; Kramer et al. 2012). For nine of the xenobiotics, the free concentrations remained constant across all three time points in both biological and stability control samples, indicating a consistent exposure of the microtissues for up to 72 h (Fig. 1a, Supplementary Figs. 9 and 10). Given the consistency between biological and stability control samples, it is unlikely that any measurable uptake into the microtissues, partitioning into cell membranes, or biotransformation occurred. In contrast, the free concentration of idarubicin, at both low and high nominal concentrations, and in stability control samples, reduced over time (Fig. 1b, Supplementary Fig. 9), indicating some chemical instability.

The observed differences between the measured and nominal exposure concentrations highlights the importance of quantifying sample-specific concentrations. Without free concentration measurements, relying on nominal exposure concentrations can lead to inaccurate descriptions of concentration–response behaviour. Incorporating quantification of free xenobiotic as part of pharmaco- or toxico-dynamic in vitro assays (using metabolomics), as presented here, enables a more accurate estimation of the biologically effective dose, facilitating an improved prediction of in vivo efficacy or toxicity, respectively (Groothuis et al. 2015).

### Xenobiotic biotransformation competency of cardiac microtissues

Biotransformation products of exposure xenobiotics formed by test systems can be elucidated using metabolomics data (Bowen et al. 2023; Sostare et al. 2024). To this end, the biotransformation capacity of cardiac microtissues was evaluated using the intracellular (DIMS) and extracellular (UHPLC-MS) metabolomics datasets acquired here. Intensity-based filters were applied to twelve peak matrices (one per xenobiotic), per analytical assay. The discovered putative xenobiotic-related features were annotated against SyGMa biotransformation predictions using BEAMSpy (MS^1^ data) and MetFrag (MS/MS data).

**Fig. 2:**
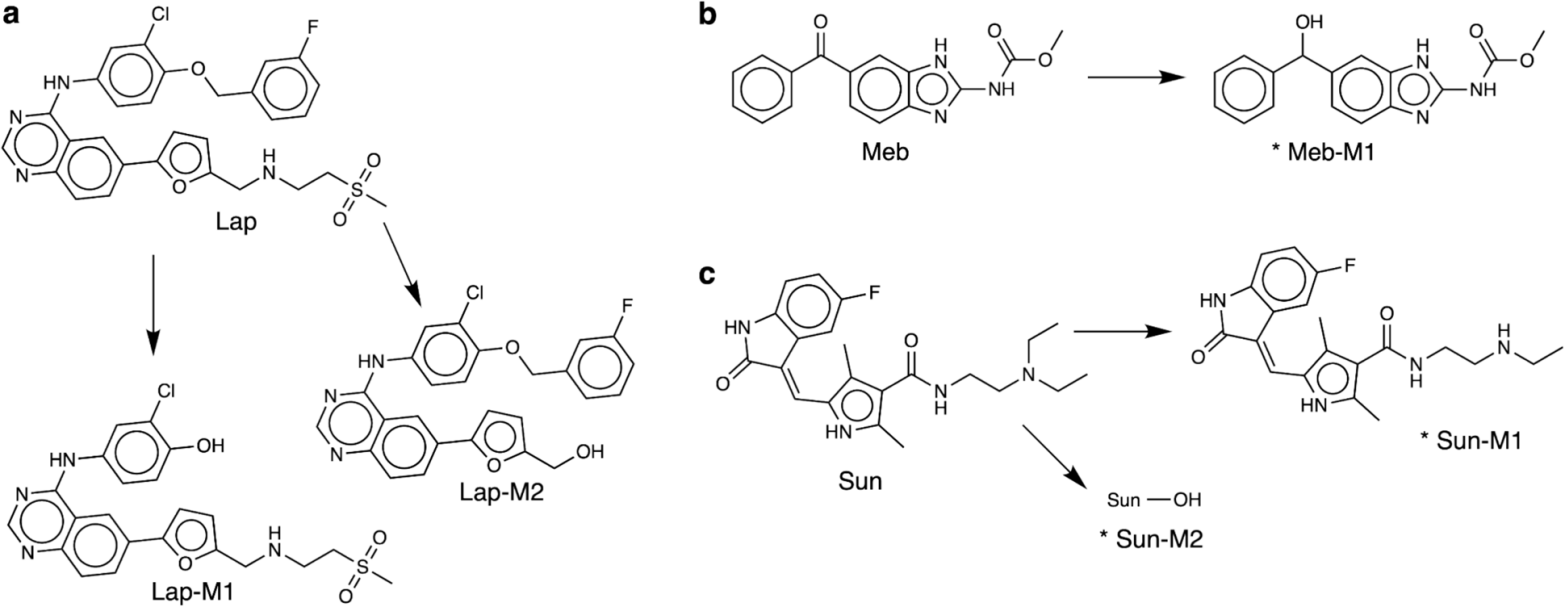
Parent xenobiotics and products of their biotransformation discovered in both intracellular extracts and spent culture medium of exposed cardiac microtissues. Shown, are the molecular structures of three parent xenobiotics and products of their biotransformation that were measured both by DIMS of the intracellular extracts and UHPLC-MS of the spent culture medium of exposed cardiac microtissues: **a** lapatinib, **b** mebendazole, and, **c** sunitinib. Arrows from the parent structures indicate routes of biotransformation, with the molecular structures of resulting biotransformation products shown. Structures annotated with * are supported by MS/MS fragmentation data (MSI level 2–3, Schymanski level 2b-3). The structural annotations of biotransformation products of lapatinib are based on MS^1^ m/z measurement only (MSI level 3–4, Schymanski level 3–4)

Two, one, two, one, and two BTPs of acyclovir, dasatinib, lapatinib, mebendazole, and sunitinib, respectively, were discovered within the spent culture media, (Supplementary Table 6). Of those, two, one, and two BTPs of lapatinib, mebendazole, and sunitinib, respectively, were also discovered within the intracellular extracts (Fig. 2). Up to 52 further BTPs, including 9, 5, 16, 2, 8, 4, 4, and 9 for clozapine, dasatinib, erlotinib, idarubicin, lapatinib, mebendazole, sorafenib, and sunitinib, respectively, were putatively discovered within the intracellular extracts (Supplementary Table 7). However, considering the absence of RT in DIMS data, which makes it infeasible to distinguish between products of analytical, i.e. ion source-based modifications, and biological transformations, care must be taken in the interpretation of these results.

Considering the metabolism required to form the BTPs discovered intracellularly and extracellularly from the parent xenobiotics, the results presented here provide strong evidence for cardiac microtissues possessing phase I metabolic capabilities, with *ca.* 90% of the BTPs reported (both putative and more confident assignments) generated by phase I metabolism (Supplementary Tables 6, 7). There is also limited evidence of phase II metabolic capability with five and one phase II BTPs of erlotinib and sorafenib, respectively, putatively discovered (Supplementary Table 7). These findings on the metabolic capability of microtissues are consistent with previously reported findings on the metabolic capability of hiPSC-CMs (Bowen et al. 2023), the most abundant of the three cell types in cardiac microtissues (Archer et al. 2018). Comparison with the earlier study revealed six of nine, and two of five sunitinib BTPs discovered intracellularly and in the spent culture medium of hiPSC-CMs, respectively, were also discovered in the current study, all of which were formed by phase I oxidative and/or hydrolytic reactions.

Taken together, the results presented here show cardiac microtissues possess some xenobiotic biotransformation competency. This is the first reported data evidencing xenobiotic biotransformation by cardiac microtissues. The ability to biotransform xenobiotics may promote the use of cardiac microtissues for predicting in vivo toxicity given the greater translational relevance of in vitro models which more closely replicate the in vivo exposure environment, where cells are typically exposed to both parent xenobiotics and their BTPs (Coecke et al. 2006).

### Exploring the metabolic responses of cardiac microtissues to xenobiotic exposure

**Fig. 3:**
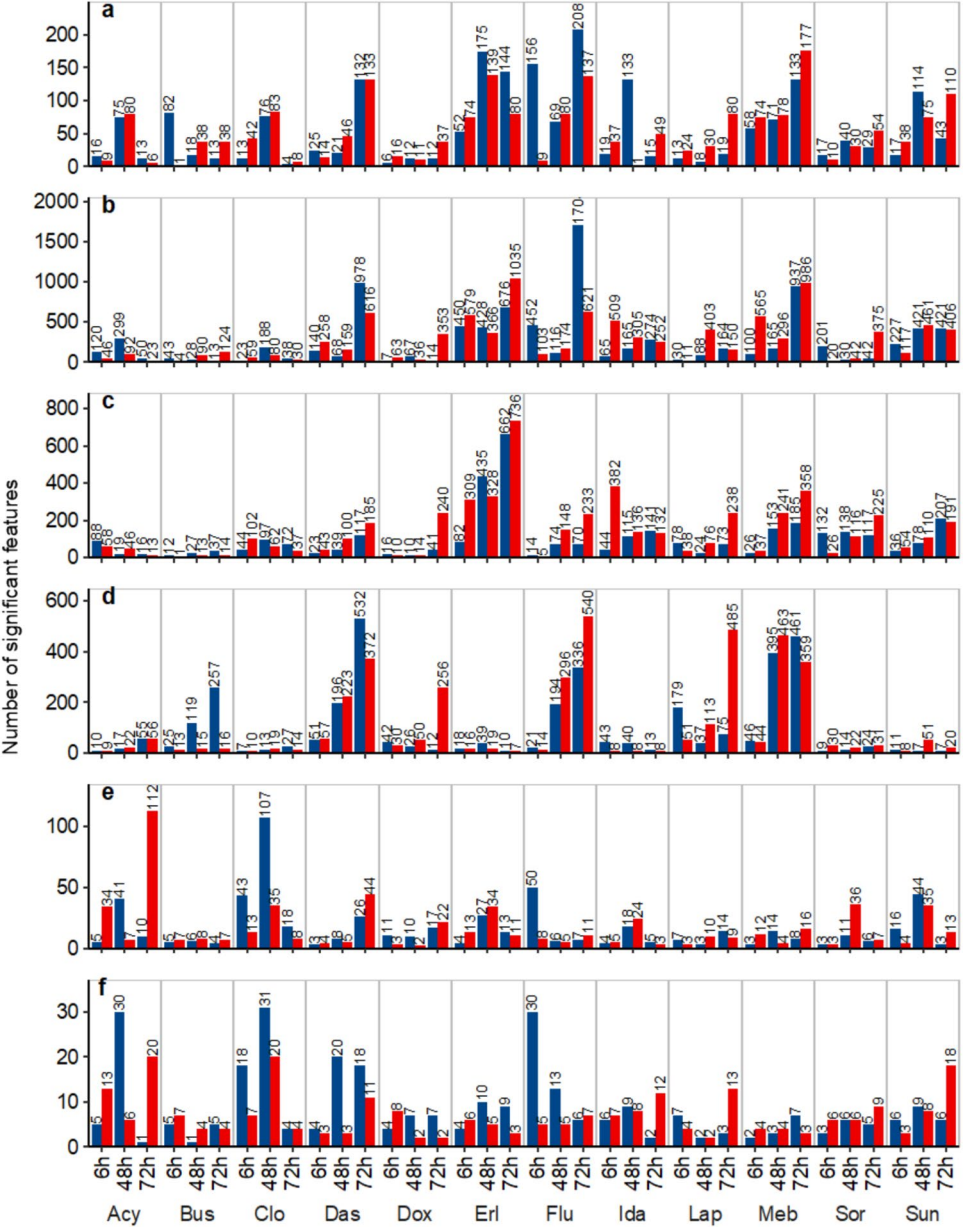
Magnitude of perturbation in cardiac microtissues induced by 72 different exposure conditions. Bar charts display the number of *m*/*z* features measured in intracellular **a**, **b** polar metabolite, and **c**, **d** lipid extracts of cardiac microtissues exposed to one of the twelve xenobiotics at either a low (blue bars) or high (red bars) concentration for 6, 48 or 72 h, with significantly different intensity relative to batch- and time-matched controls. Also shown, **e**, **f**, are the number of *m*/*z*-RT features measured in spent culture media of exposed cardiac microtissues with significantly different intensity relative to controls. Data were measured by **a** DIMS polar positive, **b** DIMS polar negative, **c** DIMS lipids positive, **d** DIMS lipids negative, **e** HILIC positive UHPLC-MS and **f** HILIC negative UHPLC-MS analytical assays. Significance was defined as *p* < 0.01, as determined by two-tailed Welch’s t-test (Color figure online)

This study was designed to investigate the response of cardiac microtissues following exposure to eight structurally cardiotoxic and four non-structurally cardiotoxic xenobiotics. The responses after 6, 48 and 72 h exposures to two phenotypically anchored concentrations of each xenobiotic (Table 1) were measured by DIMS-based untargeted metabolomics of microtissue polar metabolite and lipid intracellular extracts, and HILIC UHPLC-MS-based untargeted metabolomics of the microtissue spent culture medium. Following internal quality control measures (Supplementary Table 8, Supplementary Fig. 11), univariate statistical analysis was employed to investigate the magnitude of perturbations induced in the cardiac microtissues by each of the 72 exposure conditions (twelve xenobiotics, two concentrations, three exposure durations), relative to batch- and exposure duration-matched controls (Fig. 3). Consistent with expectations, the largest perturbation to the intracellular metabolome and lipidome was most frequently observed (57.3%) after the longest duration (72 h) exposure, followed by 48 h (28.1%). The anticipated relationship between magnitude of response and exposure concentration was less clear, with only 54.9% of cases behaving as predicted, though this can in part be explained by the quantitation of free xenobiotic concentrations in the culture media that revealed the actual differences between high and low exposure concentrations were often smaller than the nominal values (Fig. 1).

In contrast to the intracellular response, perturbation of the microtissue metabolic footprint (measured in the spent culture media) was of relatively low magnitude, with an average (per exposure condition) of just 16 and 8 *m/z*-RT positive and negative ion significant features, respectively (Fig. 3e, f). This is consistent with the metabolic footprint of in vitro models being less comprehensive in its coverage of molecular mechanisms and highlights the value of intracellular measurements in discovery-based studies.

Following putative annotation of the significant features (Supplementary Tables 9, 10), changes conserved amongst structurally cardiotoxic xenobiotics were investigated. A significant reduction in the intracellular levels of *sn*-glycero-3-phosphoethanolamine was common to structurally cardiotoxic xenobiotics. This perturbation occurred in response to either 48 or 72 h exposure to clozapine, dasatinib, fluorouracil, idarubicin, sorafenib, and sunitinib (Supplementary Fig. 12a, b). Glycero-3-phosphoethanolamine is the breakdown product of phosphatidylethanolamine, with changes in its intracellular levels conceivably a marker of disrupted glycerophospholipid metabolism. Perturbation of both Cer 33:1;O_2_ and Cer 43:2;O_2_ were also specific to structurally cardiotoxic xenobiotics, with significantly reduced intracellular levels following 72 h exposures to dasatinib, fluorouracil, lapatinib, and sunitinib (Supplementary Fig. 12c, d). This suggests these four xenobiotics may share a similar MoA involving these ceramides in the onset of SCT.

Taken together, the application of univariate analysis of untargeted metabolomics data provided insights into the magnitude of responses induced by several exposure conditions, revealing xenobiotic-, concentration-, and temporal-based trends. Perturbations to only three lipids were conserved across SCT-specific exposure conditions.

### Discovering a predictive signature of drug-induced structural cardiotoxicity

**Fig. 4:**
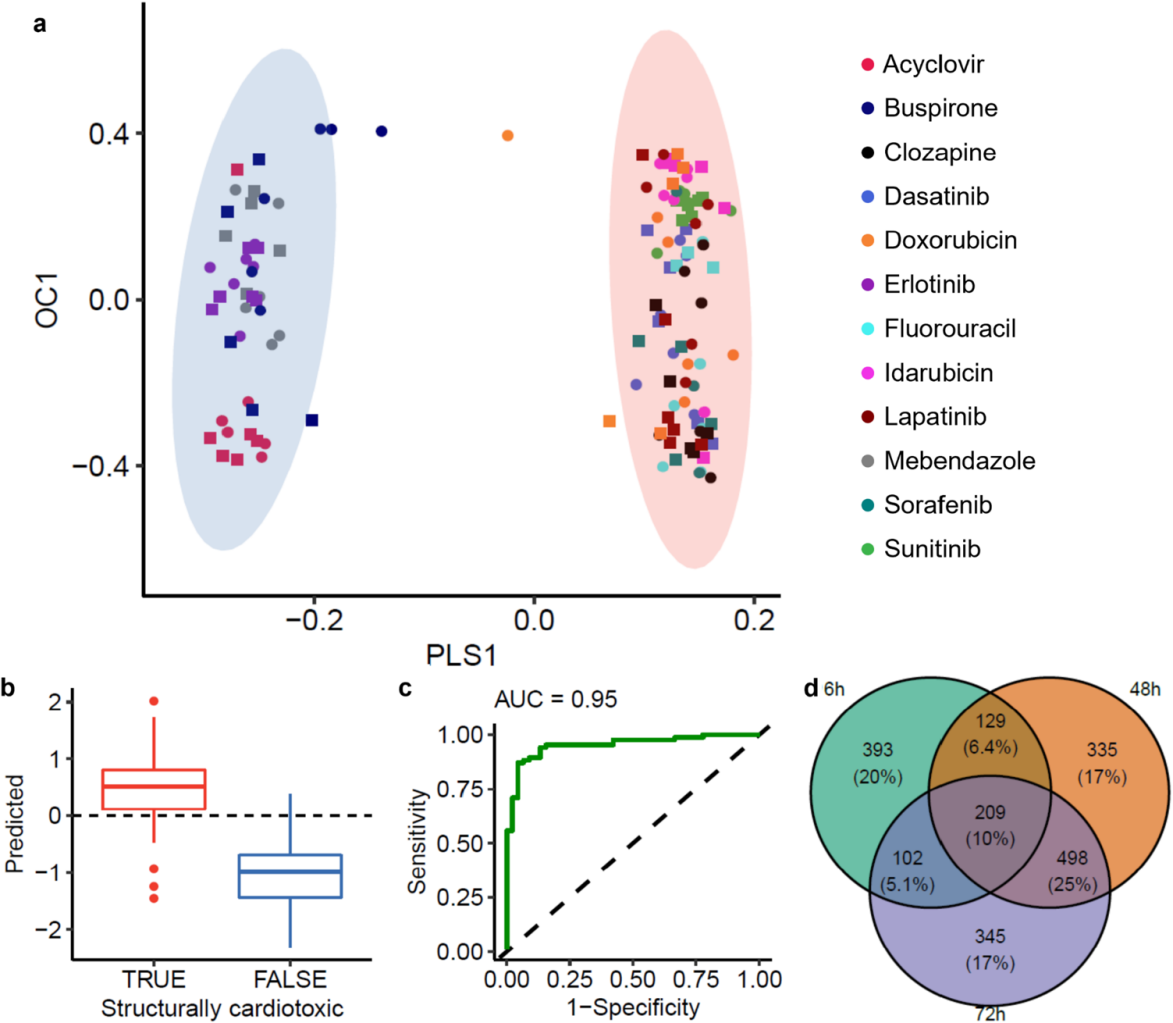
OPLS-DA model predictive of structural cardiotoxicity. **a** OPLS-DA scores plot showing the predictive (PLS1) and first orthogonal (OC1) components of a model which discriminates between the responses of cardiac microtissues induced by 48-h exposure to structurally cardiotoxic xenobiotics compared with non-structurally cardiotoxic xenobiotics. Shaded regions represent the 95% confidence regions for structurally cardiotoxic (red) and non-structurally cardiotoxic (blue) xenobiotics. Responses to both low (filled rectangle) and high (filled circle) concentrations of the twelve test xenobiotics were used for model training. **b** Boxplot of predicted values compared with true values as determined by k-fold cross validation, where a value of 1 corresponds to a sample exhibiting structural cardiotoxicity, demonstrating good predictive accuracy. c Receiver operator curve (ROC) demonstrating good specificity and sensitivity of the model in the prediction of structural cardiotoxicity, as determined by k-fold cross validation. The model shown in a-c was built from a subset of predictive features with VIP scores ≥ 1 in an initial model, measured by polar negative DIMS of cardiac microtissue intracellular extracts. Intensity measurements were corrected for batch and baseline temporal effects by normalisation against the median intensities in batch- and time-matched controls prior to analysis. d Venn diagram comparing the m/z features predictive of structural cardiotoxicity (VIP ≥ 1) from the initial models built using the entire dataset (and used to build the refined OPLS-DA models) across the three exposure durations. Data shown is from polar negative DIMS analysis of cardiac microtissue intracellular extracts (Color figure online)

Supervised multivariate analysis provides an approach to mine datasets for measured variables predictive of class labels (Quintas et al. 2021). Here, OPLS-DA was applied to mine the untargeted metabolomics datasets for metabolic and lipid features which discriminate between responses of cardiac microtissues induced by structurally cardiotoxic versus non-structurally cardiotoxic xenobiotics, for each combination of analytical assay and exposure duration (Fig. 4, Supplementary Fig. 13, Supplementary Table 11). Valid OPLS-DA models, i.e. demonstrating significantly better predictivity relative to models built after randomly permuting the input data, were built for 17 of the 18 datasets (three time points across six assays). A valid OPLS-DA model of the 6-h intracellular lipids negative data could not be built, and these data were not considered further. Comparison of the features used to build the refined predictive models revealed a conservation of > 20% (ranging 20.6–35% across four assays) of intracellular features predictive of SCT between 48 and 72 h (Fig. 4d, Supplementary Fig. 14). Commonalities in predictive features demonstrate some temporal stability of responses associated with SCT.

**Fig. 5:**
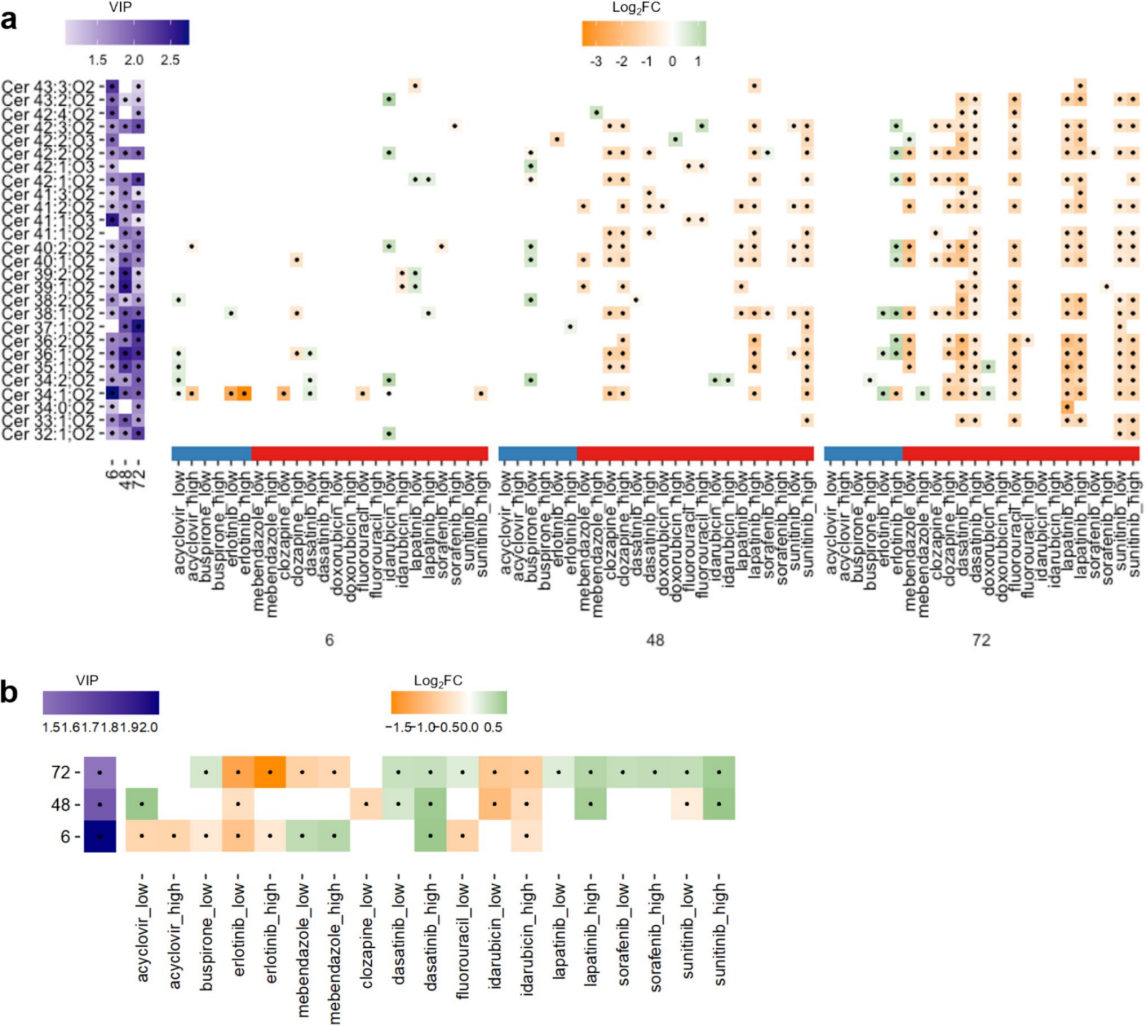
Intracellular metabolic responses predictive of the structural cardiotoxicity potential of 12 test xenobiotics. Far left of each plot shows the average VIP scores per exposure duration of **a** 27 ceramides and **b** pantothenic acid from the refined OPLS-DA models. Also shown are the significant (Welch’s t-test, *p* < 0.05) log_2_ fold changes representing the responses of a the 27 ceramides and b pantothenic acid induced by each of the exposure xenobiotics, at two concentrations (low, high) and three exposure durations (6-, 48-, and 72-h). The coloured bar on the x-axis indicates the structural cardiotoxicity label of the exposure xenobiotics (blue: not structurally cardiotoxic, red: structurally cardiotoxic) (Color figure online)

Following annotation, putative biomarkers of SCT were revealed (Supplementary Tables 12, 13). The most striking discovery was a large group of 29 intracellular ceramides (43.3% of all ceramides previously discovered in untreated cardiac microtissue extracts, Bowen et al. 2025) that are predictive of SCT (OPLS-DA), of which a total of 27 were significantly reduced by exposure to at least one of the structural cardiotoxins (Welch’s *t*-tests, Fig. 5a, Supplementary Table 9). Ceramides are integral cell membrane components which act as second messengers in cell signalling processes (Castro et al. 2014). They have been linked to multiple cardiac pathologies and are proposed clinical biomarkers of myocardial infarction and risk of death in patients with stable coronary heart disease (CERT2 risk score) (Shu et al. 2022). Furthermore, ceramides have been associated with mitochondrial dysfunction and oxidative stress, established key events of drug-induced SCT (Rocca et al. 2022; Bekhite et al. 2021; Law et al. 2018; Kretzschmar et al. 2021). Despite the evidenced association of ceramides to pathological mechanisms associated with SCT, the underlying MoA and the relationship between chain length and effect has yet to be established.

Intracellular levels of adenosine tri-, di- and mono-phosphate (ATP, ADP, and AMP, respectively) were also found to be associated with SCT (Supplementary Table 12). Moreover, AMP was measured at significantly increased levels in response to six of the tested structural cardiotoxins (Supplementary Table 9). This is consistent with the central role of cellular energetics in the onset and development of SCT (Rocca et al. 2022; Russo et al. 2021). Similarly, reduced creatine levels were found to be associated with SCT at 48 and 72 h by OPLS-DA (consistent with univariate statistical analysis; Supplementary Table 12). The creatine kinase system (the reversible synthesis of phosphocreatine and ADP from creatine and ATP, catalysed by creatine kinase) functions as a primary energy store, restoring ATP concentration at times of increased energy demand (Balestrino 2021; Del Franco et al. 2022). Perturbed creatine levels are likely indicative of a disrupted creatine kinase system and myocardial energy depletion, consistent with anthracycline-induced cardiotoxicity (Balestrino 2021). Intracellular levels of several constituents of the purine metabolism pathway (deoxyadenosine, inosine, cyclic AMP, cyclic guanosine monophosphate (GMP), GMP, adenosine/deoxyguanosine, xanthine and glutamine, in addition to AMP, ADP and ATP) were also discovered to be the predictive of SCT by OPLS-DA (Supplementary Fig. 15, Supplementary Table 12). The perturbation of these purine metabolism intermediates may also be linked to disrupted energy metabolism and the onset of SCT since a key role of purine metabolism is restoration of the adenine nucleotide pool (Zieliński et al. 2019). The intracellular levels of pantothenic acid were also found to be associated with SCT by OPLS-DA (Fig. 5b, Supplementary Table 12), consistent with univariate statistical analysis evidencing a significant increase in response to five of the tested structural cardiotoxins (Supplementary Table 9). Pantothenic acid has previously been reported as a potential biomarker of doxorubicin-induced cardiotoxicity (Wen et al. 2020; Yi et al. 2018). Via its conversion to coenzyme A, pantothenic acid has a role in fatty acyl β-oxidation, thus its levels can be indicative of (disrupted) cardiac energetics. Taken together, results shown here evidence an association between perturbed cardiac energetics and drug-induced SCT and demonstrate metabolic biomarkers of cardiac energy status are suitable for predicting SCT. Other putatively annotated predictive features discovered by OPLS-DA include reduced and oxidised glutathione (Supplementary Table 12). Moreover, reduced glutathione was measured at significantly decreased levels following exposure to three of the tested structural cardiotoxins: dasatinib, fluorouracil and sunitinib. The evidenced predictive capacity of intracellular glutathione levels is consistent with the established association of oxidative stress with SCT (Bouitbir et al. 2019; Songbo et al. 2019).

Considering features measured within the metabolic footprint of cardiac microtissues, three of four metabolites previously selected for a targeted metabolomics screening assay to predict structural and functional cardiotoxicity from measurements of spent culture media of hiPSC-CMs (Palmer et al. 2020) were also discovered to predict SCT here by OPLS-DA: lactic acid (6- and 48-h), thymidine (48 and 72 h), and deoxycytidine (6, 48 and 72 h) (Supplementary Table 13). Furthermore, additional metabolites, L-alanine (72 h) and N-acetylaspartic acid (6, 48 and 72 h), found to be predictive of cardiotoxicity but not included in the final assay panel by Palmer et al. (2020), were similarly discovered to be predictive in this study. Our approach discovered an extracellular predictive signature containing many additional metabolites, e.g. inosine and cytosine, which had high VIP scores at 6, 48 and 72 h (Supplementary Table 13).

Overall, OPLS-DA of untargeted metabolomics data measuring responses of cardiac microtissues to eight structurally cardiotoxic and four non-structurally cardiotoxic xenobiotics, at two concentrations, after 6, 48, and 72 h, has discovered *m/z* and *m/z-*RT features with capacity to predict SCT. Represented amongst the panel of predictive features (Table 2) are putatively annotated metabolites associated with myocardial energy status (ATP, ADP, AMP, creatine, purine metabolism pathway constituents, and pantothenic acid). The predictive metabolic signature also includes putatively annotated markers of mitochondrial oxidative stress, mainly reduced and oxidised glutathione. These findings are consistent with established mechanisms of SCT. The inclusion of many ceramides in the predictive signature evidences a role for these lipids in the onset of SCT. A mechanism explaining this association is less established. Taken together, the evidence provided by the putatively annotated metabolite and lipid signatures discovered here suggests the prediction of SCT may be achieved through a biomarker panel that combines previously known and currently undefined mechanisms of SCT.

**Table 2:** Summary of the discovered metabolic signature predictive of drug-induced structural cardiotoxicity in cardiac microtissues

Biochemical function	Metabolites/Lipids	Evidence
Ceramide signalling	Cer 32:1; O2 | Cer 33:1; O2 | Cer 34:0; O2 | Cer 34:1; O2 | Cer 34:2; O2 | Cer 35:1; O2 | Cer 36:1; O2 | Cer 36:2; O2 | Cer 37:1; O2 | Cer 38:1; O2 | Cer 38:2; O2 | Cer 39:1; O2 | Cer 40:1; O2 | Cer 40:2; O2 | Cer 41:1; O2 | Cer 41:1; O3 | Cer 41:2; O2 | Cer 41:3; O2 | Cer 42:1; O2 | Cer 42:1; O3 | Cer 42:2; O2 | Cer 42:2; O3 | Cer 42:3; O2 | Cer 42:4; O2 | Cer 43:2; O2 | Cer 43:3; O2	OPLS-DA (VIP score) Significantly reduced levels in response to structural cardiotoxins (Supplementary Table 9) Consistent with previous findings of significant perturbation of ceramides in rat heart and plasma following treatment with sunitinib or KU60648 (Bowen et al. 2023)
Energy metabolism	Creatine	OPLS-DA (VIP score) Significantly increased levels of AMP and pantothenic acid, and significantly reduced levels of glutathione observed in response to structural cardiotoxins (Supplementary Table 9) Consistent with reports of an association between anthracycline-induced cardiotoxicity and disrupted creatine kinase system (Balestrino 2021) Consistent with the previous association of structural cardiotoxicity with perturbed purine metabolism (Bowen et al. 2021) Consistent with previous reports of pantothenic acid as biomarker of doxorubicin-induced cardiotoxicity (Wen et al. 2020; Yi et al. 2018) Reduced/oxidised glutathione are established markers of mitochondrial oxidative stress, a phenotype of structural cardiotoxicity (Bouitbir et al. 2019; Songbo et al. 2019)
	L-glutamine	
	Deoxyadenosine	
	Inosine	
	Cyclic AMP	
	Cyclic GMP	
	GMP	
	Adenosine	
	Deoxyguanosine	
	ATP	
	ADP	
	AMP	
	Pantothenic acid	
	Reduced/Oxidised glutathione	
Extracellular markers	Lactic acid	OPLS-DA (VIP score) Consistent with findings of Palmer et al. (2020)
	Thymidine	
	Deoxycytidine	
	L-alanine	
	N-acetylaspartic acid	

Metabolites and lipids, annotated to MSI level 1 or 2, found to be predictive of structural cardiotoxicity by OPLS-DA analysis, and supported by published literature, are reported, grouped by broad biochemical function

## Conclusions

In summary, extensive untargeted metabolomics data measuring the fate of eight structurally cardiotoxic and four non-structurally cardiotoxic xenobiotics and the effects they induced on the intracellular metabolome, intracellular lipidome and metabolic footprint of cardiac microtissues after 6, 48, and 72 h exposure to two concentrations were acquired. Quantitation of the xenobiotics in the spent culture medium revealed free concentrations to which the microtissues were exposed were typically lower than the nominal concentrations. This highlights the importance of these measurements in in vitro assays, where data are extrapolated to predict clinical toxicity or efficacy, with the use of measured free concentrations expected to improve prediction accuracy compared to nominal concentrations. Meanwhile, the discovery of xenobiotic BTPs both intracellularly and within the spent culture medium provided the first direct evidence of biotransformation competency of cardiac microtissues. The characterisation of the biotransformation in test systems is often overlooked, despite its potential to improve the accuracy of IVIVE and safety predictions. The demonstrated biotransformation capability underscores the high translational relevance of cardiac microtissues.

Investigation into the effects of xenobiotic exposure revealed concentration-dependent and temporal trends in the magnitude of perturbation induced by each xenobiotic and revealed few responses specific and common to structural cardiotoxin exposure. Supervised multivariate analysis was employed to mine the data for features predictive of SCT. Multiple significantly predictive OPLS-DA models were built for each assay and exposure duration combination, discovering signatures within the metabolic and lipid fingerprints, as well as the metabolic footprint, capable of predicting the cardiotoxicity classification of the test xenobiotics. Annotation of these signatures revealed consistencies with previous findings, anchoring them to established MoAs, such as perturbed creatine kinase systems and disrupted purine metabolism, whilst also providing evidence to hypothesise novel MoAs, such as the role of ceramides in the onset of SCT.

Future efforts to refine and validate the panel are now required towards more accurate prediction of SCT during the early stages of drug discovery. The availability of free xenobiotic exposure concentrations and a better understanding of the extent of xenobiotic biotransformation in cardiac microtissues will support these future efforts, particularly in translating findings across test systems.

## Supplementary Information

Below is the link to the electronic supplementary material.

Supplementary file1 (DOCX 5263 KB)

Supplementary file2 (XLSX 721 KB)

## Data Availability

The datasets from the current study are available from the corresponding author upon reasonable request.
