# Supplementary material for: Discovering a predictive metabolic signature of drug-induced structural cardiotoxicity in cardiac microtissues
Source: Arch Toxicol. 2025 May 16;99(8):3379–92. doi: 10.1007/s00204-025-04074-4 (PMC12367874; doi:10.1007/s00204-025-04074-4)
Supplement: Supplementary file 1 — Supplementary file1 (DOCX 5263 KB) [file 204_2025_4074_MOESM1_ESM.docx]

# Supplementary Information

## Discovering a predictive metabolic signature of drug-induced structural cardiotoxicity in cardiac microtissues

Tara J. Bowen^1†^, Andrew R. Hall^2^, Gavin R. Lloyd^3^, Matthew J. Smith^3^, Ralf J. M. Weber^1,3^, Amanda Wilson^4^, Amy Pointon^2^, Mark R. Viant^1,3,^*

^1^ School of Biosciences, University of Birmingham, Edgbaston, Birmingham, B15 2TT, UK

^2^ Safety Sciences, Clinical Pharmacology and Safety Sciences, BioPharmaceuticals R&D, AstraZeneca, Cambridge, UK

^3^ Phenome Centre Birmingham, University of Birmingham, Edgbaston, Birmingham, B15 2TT, UK

^4^ Integrated Bioanalysis, Clinical Pharmacology and Safety Sciences, BioPharmaceuticals R&D, AstraZeneca, Cambridge, UK

^†^ Current affiliation: Medicines Discovery Catapult, Alderley Park, Cheshire, SK10 4TG, UK

* Corresponding author - [m.viant@bham.ac.uk](mailto:m.viant@bham.ac.uk)

##

## **Supplementary Methods**

### **Selection criteria for exposure xenobiotics**

To control, to some extent, against analytical variation induced by ion suppression and competition effects, which depend on the ionisation efficiency of the exposure xenobiotics, the list of 30 xenobiotics was filtered to contain only basic compounds. Related, structurally cardiotoxic xenobiotics with very low potency according to high content biology (HCB) data were filtered out since exposing at higher concentrations increases the likelihood of analytical challenges caused by the xenobiotics. A ratio of 1:2 non-structurally cardiotoxic/structurally cardiotoxic xenobiotics was chosen, such that there were sufficient structurally cardiotoxic xenobiotics to discover a relatively generalisable and consistent metabolic signature while having a sufficient ‘out group’ to reliably assess specificity.

Further criteria to select the eight structural cardiotoxic xenobiotics included:

1. Xenobiotics must be clinically recognised structural cardiotoxins, i.e., U.S. Food and Drug Administration (FDA) approval label must include structural-based cardiovascular warnings.

2. Include subsets of xenobiotics which induced similar *in vitro* responses while maintaining diversity (based on hierarchical clustering analysis (HCA) of HCB measurements, Supplementary Fig. 1a).

3. Maintain chemical class diversity – include at least one anthracycline and one tyrosine kinase inhibitor (TKi).

Further criteria to select the four non-structurally cardiotoxic xenobiotics included:

1. Xenobiotics do not exhibit cardiotoxicity by any mechanism, i.e., no cardiovascular warnings on the FDA approval label.

2. Xenobiotics must not induce a response against HCB indicators at therapeutically relevant concentrations (<10x C_max_, the maximum clinical blood concentration following treatment at therapeutic dose)

3. Include xenobiotics of the same chemical class or with structural similarity to the selected structurally cardiotoxic xenobiotics.

4. Maintain diversity in terms of *in vitro* response (based on HCA of HCB measurements –Supplementary Fig. 1b).


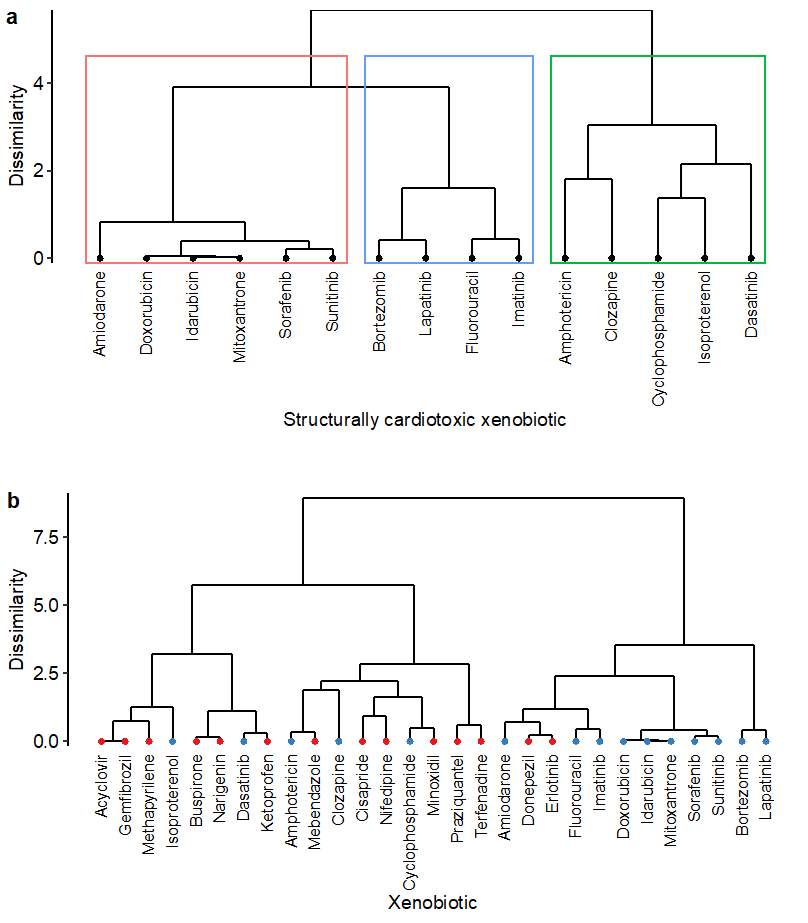


**Supplementary Fig. 1 (Dis)similarity of the cardiac microtissue response to xenobiotics.**The dendrograms show the dissimilarity of the cardiac microtissues response following exposure to **a** 15 structurally cardiotoxic xenobiotics and **b** 29 xenobiotics, 15 structurally cardiotoxic (blue) and 14 non-structurally cardiotoxic (red), for 72-hours. Responses were defined by three parameters: % ATP depletion, % mitochondrial membrane permeability (Δψm) and % endoplasmic reticulum (ER) stress, measured by high content biology. The dissimilarity matrices were calculated using the Euclidean distance metric and clustering performed using Ward's method. Coloured boxes in **a** indicate three separate clusters of xenobiotics which induce highly similar responses in cardiac microtissues: the red box highlights xenobiotics which present relatively high potency against all three indicators, the blue box highlights xenobiotics which present relatively high potency against Δψm and ATP indicators, but relatively low potency according to % ER stress, and the green box highlights xenobiotics which present relatively low potency against all three indicators.

### **Resuspension of microtissue extracts**

Resuspension of dried extracts was performed using a Biomek i7 Hybrid Workstation (i7, Beckman Coulter). Following addition of resuspension solvent (either 30 μL 4:1 (v/v) methanol/water with 0.25% formic acid, 30 μL 4:1 (v/v) methanol/25 mM aqueous ammonium acetate, or 40 μL 2:1 (v/v) 7.5 mM methanolic ammonium acetate/chloroform), microplates were placed on the orbital shaker and shaken for 1-minute at 300 revolutions per minute (rpm), then spun in a centrifuge for 3 mins (3,622-*g­* at 4℃). 20 or 30 μL polar or lipid resuspended extracts, respectively, were then transferred to a 384-well Eppendorf twin-tec analysis plate. The analysis plate was sealed with heat-seal foil, centrifuged at 2,000-*g* for 10 mins (4℃), then placed in a TriVersa Nanomate sample compartment, maintained at 10℃, for analysis.

### **Modified SIM-stitching nESI-DIMS^(n)^**

The newly developed spectral stitching method implemented in this study consisted of either 19 or 33 selected ion monitoring (SIM) windows of variable width for analysis of the polar metabolites and lipids, respectively (Supplementary Tables 1 and 2). The widths of the SIM windows were defined using existing mass spectral measurements from cardiac microtissue intracellular extracts (Bowen et al., 2021) for each of the four assays separately. Specifically, SIM windows were defined such that the total ion population was evenly distributed between SIM windows (Kang et al., 2019, Sarvin et al., 2020). Furthermore, the size of the overlapping mass range between adjacent SIM windows was reduced from 10 Da to 5 Da, accounting for the improved ion isolation efficiency profile offered by the ID-X quadrupole compared with the Orbitrap Elite ion trap (original spectral stitching method).

***Supplementary Table 1 Spectral stitching nESI-DIMS method parameters.*** *Mass spectrometry parameters for the acquisition of untargeted metabolomics data by four spectral stitching-based nESI-DIMS assays performed on an Orbitrap ID-X Tribrid mass spectrometer.*

| Assay | Polar positive | Polar negative | Lipids positive | Lipids negative |
| --- | --- | --- | --- | --- |
| Ionisation mode | Positive | Negative | Positive | Negative |
| Full mass range | 50 – 620 | 50 – 620 | 190 – 2000 | 190 – 2000 |
| Number of SIM windows | 19 | 19 | 33 | 33 |
| Number of internal replicates | 3 | 3 | 3 | 3 |
| Approximate method length (min) | 3.9 | 3.9 | 3.9 | 3.9 |
| Ion transfer tube temperature (°C) | 200 | 200 | 200 | 200 |
| RF lens voltage (%) | 30 | 30 | 60 | 60 |
| Isolation mode | Quadrupole | Quadrupole | Quadrupole | Quadrupole |
| Isolation window width | Variable | Variable | Variable | Variable |
| Mass analyser | Orbitrap | Orbitrap | Orbitrap | Orbitrap |
| Number of microscans | 7 | 7 | 4 | 4 |
| Resolution | 240,000 | 240,000 | 240,000 | 240,000 |
| AGC target | 5x10^4^ | 5x10^4^ | 5x10^4^ | 5x10^4^ |
| Maximum injection time (ms) | 200 | 200 | 200 | 200 |

***Supplementary Table 2 SIM window definitions for nESI-DIMS-based untargeted metabolomics.*** *The m/z ranges and widths of SIM windows employed in the nESI-DIMS-based untargeted metabolomics assays performed using an Orbitrap ID-X Tribrid mass spectrometer.*

| Assay | SIM window ID | Start *m/z* | End *m/z* | SIM window width (Da) |
| --- | --- | --- | --- | --- |
| Polar positive | PP-SIM-1 | 50 | 125 | 75 |
| Polar positive | PP-SIM-2 | 115 | 150 | 35 |
| Polar positive | PP-SIM-3 | 140 | 165 | 25 |
| Polar positive | PP-SIM-4 | 155 | 190 | 35 |
| Polar positive | PP-SIM-5 | 180 | 210 | 30 |
| Polar positive | PP-SIM-6 | 200 | 235 | 35 |
| Polar positive | PP-SIM-7 | 225 | 255 | 30 |
| Polar positive | PP-SIM-8 | 245 | 285 | 40 |
| Polar positive | PP-SIM-9 | 275 | 315 | 40 |
| Polar positive | PP-SIM-10 | 305 | 340 | 35 |
| Polar positive | PP-SIM-11 | 330 | 365 | 35 |
| Polar positive | PP-SIM-12 | 355 | 380 | 25 |
| Polar positive | PP-SIM-13 | 370 | 395 | 25 |
| Polar positive | PP-SIM-14 | 385 | 415 | 30 |
| Polar positive | PP-SIM-15 | 405 | 435 | 30 |
| Polar positive | PP-SIM-16 | 425 | 460 | 35 |
| Polar positive | PP-SIM-17 | 450 | 490 | 40 |
| Polar positive | PP-SIM-18 | 480 | 555 | 75 |
| Polar positive | PP-SIM-19 | 545 | 620 | 75 |
| Polar negative | PN-SIM-1 | 50 | 120 | 70 |
| Polar negative | PN-SIM-2 | 110 | 145 | 35 |
| Polar negative | PN-SIM-3 | 135 | 160 | 25 |
| Polar negative | PN-SIM-4 | 150 | 195 | 45 |
| Polar negative | PN-SIM-5 | 185 | 220 | 35 |
| Polar negative | PN-SIM-6 | 210 | 255 | 45 |
| Polar negative | PN-SIM-7 | 245 | 270 | 25 |
| Polar negative | PN-SIM-8 | 260 | 285 | 25 |
| Polar negative | PN-SIM-9 | 275 | 300 | 25 |
| Polar negative | PN-SIM-10 | 290 | 320 | 30 |
| Polar negative | PN-SIM-11 | 310 | 340 | 30 |
| Polar negative | PN-SIM-12 | 330 | 370 | 40 |
| Polar negative | PN-SIM-13 | 360 | 390 | 30 |
| Polar negative | PN-SIM-14 | 380 | 405 | 25 |
| Polar negative | PN-SIM-15 | 395 | 425 | 30 |
| Polar negative | PN-SIM-16 | 415 | 465 | 50 |
| Polar negative | PN-SIM-17 | 455 | 500 | 45 |
| Polar negative | PN-SIM-18 | 490 | 555 | 65 |
| Polar negative | PN-SIM-19 | 545 | 620 | 75 |
| Lipids positive | LP-SIM-2 | 190 | 225 | 35 |
| Lipids positive | LP-SIM-2 | 215 | 245 | 30 |
| Lipids positive | LP-SIM-3 | 235 | 260 | 25 |
| Lipids positive | LP-SIM-4 | 250 | 280 | 30 |
| Lipids positive | LP-SIM-5 | 270 | 305 | 35 |
| Lipids positive | LP-SIM-6 | 295 | 320 | 25 |
| Lipids positive | LP-SIM-7 | 310 | 345 | 35 |
| Lipids positive | LP-SIM-8 | 335 | 360 | 25 |
| Lipids positive | LP-SIM-9 | 350 | 375 | 25 |
| Lipids positive | LP-SIM-10 | 365 | 405 | 40 |
| Lipids positive | LP-SIM-11 | 395 | 420 | 25 |
| Lipids positive | LP-SIM-12 | 410 | 440 | 30 |
| Lipids positive | LP-SIM-13 | 430 | 460 | 30 |
| Lipids positive | LP-SIM-14 | 450 | 480 | 30 |
| Lipids positive | LP-SIM-15 | 470 | 495 | 25 |
| Lipids positive | LP-SIM-16 | 485 | 520 | 35 |
| Lipids positive | LP-SIM-17 | 510 | 545 | 35 |
| Lipids positive | LP-SIM-18 | 535 | 575 | 40 |
| Lipids positive | LP-SIM-19 | 565 | 600 | 35 |
| Lipids positive | LP-SIM-20 | 590 | 625 | 35 |
| Lipids positive | LP-SIM-21 | 615 | 655 | 40 |
| Lipids positive | LP-SIM-22 | 645 | 680 | 35 |
| Lipids positive | LP-SIM-23 | 670 | 710 | 40 |
| Lipids positive | LP-SIM-24 | 700 | 730 | 30 |
| Lipids positive | LP-SIM-25 | 720 | 745 | 25 |
| Lipids positive | LP-SIM-26 | 735 | 760 | 25 |
| Lipids positive | LP-SIM-27 | 750 | 780 | 30 |
| Lipids positive | LP-SIM-28 | 770 | 795 | 25 |
| Lipids positive | LP-SIM-29 | 785 | 815 | 30 |
| Lipids positive | LP-SIM-30 | 805 | 840 | 35 |
| Lipids positive | LP-SIM-31 | 830 | 960 | 130 |
| Lipids positive | LP-SIM-32 | 950 | 1080 | 130 |
| Lipids positive | LP-SIM-33 | 1070 | 1200 | 130 |
| Lipids negative | LN-SIM-1 | 190 | 215 | 25 |
| Lipids negative | LN-SIM-2 | 205 | 230 | 25 |
| Lipids negative | LN-SIM-3 | 220 | 245 | 25 |
| Lipids negative | LN-SIM-4 | 235 | 260 | 25 |
| Lipids negative | LN-SIM-5 | 250 | 275 | 25 |
| Lipids negative | LN-SIM-6 | 265 | 290 | 25 |
| Lipids negative | LN-SIM-7 | 280 | 305 | 25 |
| Lipids negative | LN-SIM-8 | 295 | 320 | 25 |
| Lipids negative | LN-SIM-9 | 310 | 335 | 25 |
| Lipids negative | LN-SIM-10 | 325 | 355 | 30 |
| Lipids negative | LN-SIM-11 | 345 | 380 | 35 |
| Lipids negative | LN-SIM-12 | 370 | 400 | 30 |
| Lipids negative | LN-SIM-13 | 390 | 425 | 35 |
| Lipids negative | LN-SIM-14 | 415 | 450 | 35 |
| Lipids negative | LN-SIM-15 | 440 | 485 | 45 |
| Lipids negative | LN-SIM-16 | 475 | 505 | 30 |
| Lipids negative | LN-SIM-17 | 495 | 530 | 35 |
| Lipids negative | LN-SIM-18 | 520 | 545 | 25 |
| Lipids negative | LN-SIM-19 | 535 | 570 | 35 |
| Lipids negative | LN-SIM-20 | 560 | 585 | 25 |
| Lipids negative | LN-SIM-21 | 575 | 600 | 25 |
| Lipids negative | LN-SIM-22 | 590 | 615 | 25 |
| Lipids negative | LN-SIM-23 | 605 | 630 | 25 |
| Lipids negative | LN-SIM-24 | 620 | 645 | 25 |
| Lipids negative | LN-SIM-25 | 635 | 660 | 25 |
| Lipids negative | LN-SIM-26 | 650 | 695 | 45 |
| Lipids negative | LN-SIM-27 | 685 | 725 | 40 |
| Lipids negative | LN-SIM-28 | 715 | 755 | 40 |
| Lipids negative | LN-SIM-29 | 745 | 800 | 55 |
| Lipids negative | LN-SIM-30 | 790 | 850 | 60 |
| Lipids negative | LN-SIM-31 | 840 | 960 | 120 |
| Lipids negative | LN-SIM-32 | 950 | 1080 | 130 |
| Lipids negative | LN-SIM-33 | 1070 | 1200 | 130 |

MS^n^ data were acquired using methods based on the spectral stitching full scan (MS^1^) methods. For acquisition of MS^n^ data of endogenous features, the full mass ranges for polar or lipid assays were divided into three segments, each to be analysed by a separate sample injection. MS^1^ data were acquired by SIM scans of the same mass ranges as the corresponding full scan method (Supplementary Table 2). After each SIM MS^1^ scan, MS^n^ data were acquired for a defined period of time: either 4- or 2-mins for polar and lipid assays, respectively, before analysis progressed to the next SIM MS^1^ scan. Precursors for fragmentation were selected from measurements made in the preceding SIM MS^1^ scan, guided by a user-defined target inclusion list of toxicologically relevant metabolites (MTox700+ biomarker list, Sostare et al., 2022) and exclusion list of *m/z* features measured in a solvent blank sample. MS^n^ data acquired included higher energy collisional dissociation (HCD) MS^2^, using either three stepped normalised collision energies (CEs) or single normalised CE selected by ‘Assisted CE’, collision-induced dissociation (CID) MS^2^, and CID MS^3^ for the three most intense fragments measured by single CE HCD MS^2^ and, separately, for three most intense fragments measured by CID MS^2^ (Supplementary Fig. 2). For acquisition of MS^n^ data corresponding to xenobiotic features, the same methods were implemented except shorter 1-minute or 30-second scan cycles for each SIM window in polar and lipid assays, respectively, to allow acquisition of data across the full mass ranges from a single sample injection.


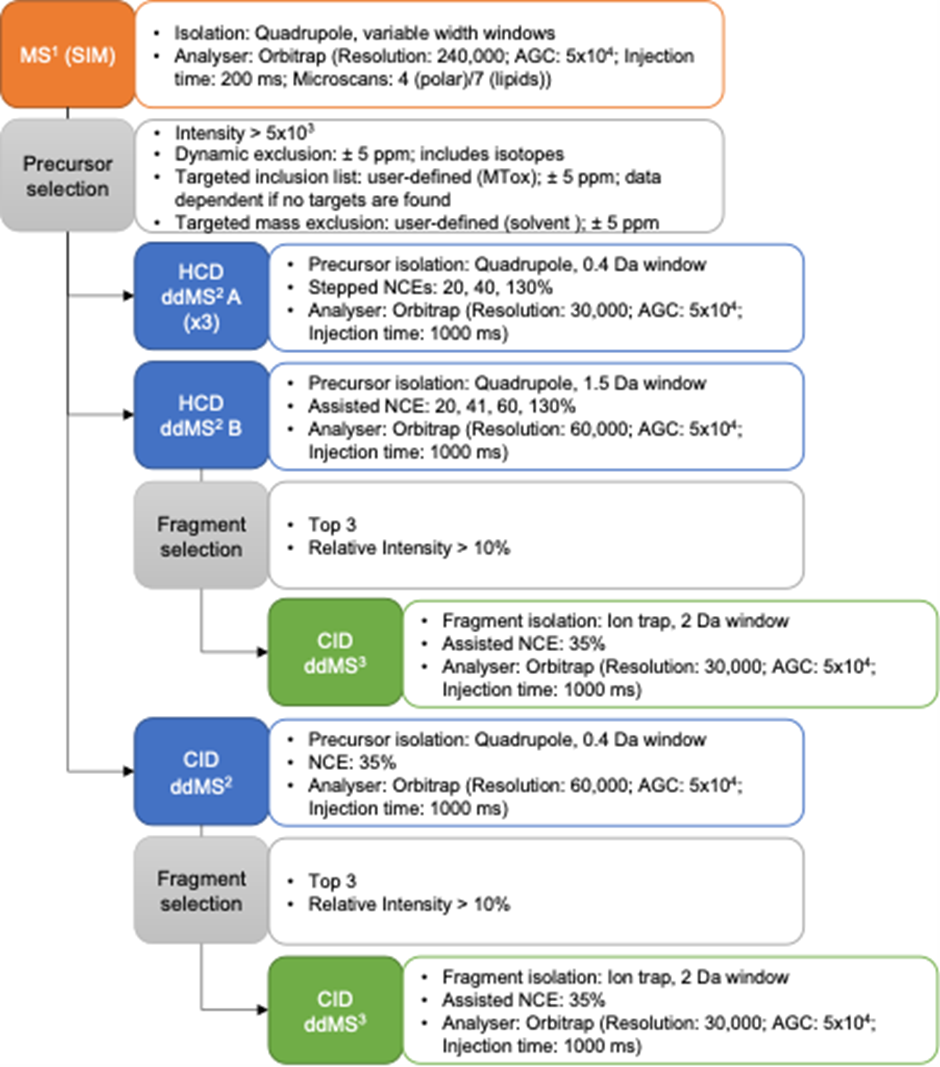


***Supplementary Fig. 2 Flow chart describing the method for acquiring MS^n^ data by nESI-DIMS using an Orbitrap ID-X Tribrid mass spectrometer.*** *Each scan cycle begins with SIM (MS^1^) scan by the Orbitrap, measurements from which are used to select precursors which meet defined criteria for subsequent fragmentation. Each selected precursor is analysed by SIM MS^1^ scan, using an isolation window of 0.4 Da before fragmentation by higher-energy collisional dissociation (HCD) at three stepped normalised collision energies (NCE): 20, 40 and 130% in triplicate. The selected precursor is also fragmented by HCD at a single, optimal NCE, selected by ‘Assisted CE’ from a choice of 20, 41, 60 and 130%. Thirdly, the selected precursor is fragmented by collision-induced dissociation (CID) in the high-pressure cell of the dual-pressure linear ion trap using an NCE 35%. All MS^2^ data are detected by the Orbitrap. Additionally, the three most intense MS^2^ fragments resulting from HCD at the single, optimal, NCE, and the three most intense MS^2^ fragments resulting from CID at NCE 35% undergo CID fragmentation, provided they pass the defined intensity filter. The resulting MS^3^ fragments are detected by the Orbitrap. This cycle is repeated for as many MS^1^ precursors as possible within the 4- or 2-minute cycle time for polar and lipid assays, respectively, before proceeding to the next SIM MS^1^ scan, at which point the cycle repeats.*

### **DIMS(^n^) data processing**

MS^1^ -based peak picking was performed over two rounds. An initial round of DIMSpy peak picking, spectral stitching and sample alignment, intra-study QCs and study samples was used to reveal outlying study samples - samples for which internal standard intensity was outside defined thresholds (median ± 3x median absolute deviation (MAD), calculated separately for intra-study QCs and study samples per analytical plate) were removed. Additionally, features corresponding to the [M+H]^+^ and [M-H]^-^ ion forms of L-tryptophan-d5-indole, and the [M+H]^+^ and [M+acetate]^-^ ion forms of dodecylphosphorylcholine-d38 were used for the polar positive, polar negative, lipids positive and lipids negative datasets, respectively. Following removal of outliers from the dataset, data were re-processed, including peak picking and spectral stitching (SNR>3, ±2 ppm mass tolerance, ≥2 internal replicates), sample alignment (±2 ppm mass tolerance), blank subtraction (features with intensity <10x median blank intensity in ≤80% non-blank samples removed) and sample filtering (features present in ≤50% non-blank samples were removed).

The following filtering and preprocessing was applied to the peak matrices prior to statistical analysis: (1) putative xenobiotic-related features (identified by the specialised workflow, see “Discovery and annotation of xenobiotic-related features”), (2) features corresponding to the internal standard, (3) features present in <50% of all non-blank samples, (4) samples with >50% missing values, and (5) features present in <70% intra-study QCs were all removed from the peak matrices. Data were then normalised by probabilistic quotient normalisation, applied to subsets of features measured by each SIM window separately. Subsequently, any features with RSD across intra-study QCs >30% after normalisation were removed.

DIMS^n^ data were processed using the python packaged MSnPy (<https://github.com/computational-metabolomics/msnpy>) as follows: (1) grouping of MS^n^ scans according to MS^1^ precursor, i.e., grouping of stepped normalised collision energy (-CE) higher-energy collisional dissociation (HCD) MS^2^, single normalised-CE HCD MS^2^, collision-induced dissociation (CID) MS^2^, and CID MS^3^ spectra, (2) peak picking across all grouped scans using signal-to-noise ratio (SNR) threshold of 10, mass tolerance of ±5 ppm, and 50% filter, where replicate scans were available, and (3) processed, grouped spectra were used to create database of spectral trees. Spectral matching was performed on each MS^2^ spectra from each spectral tree separately after conversion to msp format using msPurity (Lawson et al., 2017), with ±10 ppm mass tolerance for matching product ions.

Outputs of MSnPy processing and MS^2^ spectral matching for each data file were filtered to remove any matches where the theoretical precursor *m/*z was outside the isolation window (± 0.2 Da) used by the instrument and to retain only matches where either the dot product or reverse dot product score was >0.5. Where there were multiple compound annotations per spectrum, filtered outputs were collapsed to a single record per spectrum. Annotations were then matched to features of the MS^1^ peak-intensity matrix where the theoretical precursor *m/z* was within ±2 ppm of MS^1^ feature’s measured *m/z*, with multiple annotations originating from separate raw data files collapsed into single records per feature.

### **Batch correction**

Samples were generated, prepared, and analysed in four batches, with each batch composed of 21 exposure conditions: exposure to two structurally cardiotoxic xenobiotics, and one non-structurally cardiotoxic xenobiotic, each at two concentrations, and exposure to 0.1% DMSO (controls), for 6-, 48- and 72-hours. Data from all four batches were processed simultaneously to produce a single peak-intensity matrix per analytical assay, to ensure alignment of *m/z* or *m/z*-RT features across batches, enabling later inter-batch comparisons.

Subsequent assessment of the processed data revealed inter-batch variation, as evidenced by total ion intensity measurements and PCA (Supplementary Fig. 3a). Further interrogation of the data suggests the inter-batch variation was of a biological origin, with batch correction algorithms using intra-study QCs failing to correct the effect (Supplementary Fig. 3b). To overcome this challenge, all peak matrix filtering, normalisation, and other processing were performed per batch.


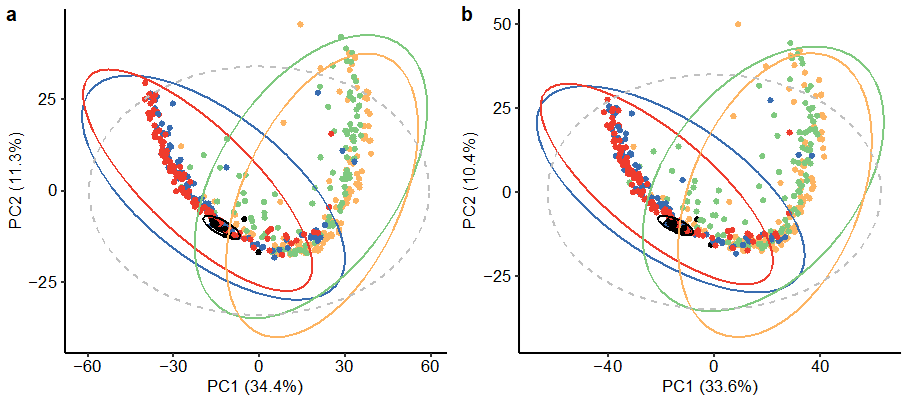


***Supplementary Fig. 3 Inter-batch variation in processed metabolomics data.*** *PCA scores plots demonstrating variation between the four exposure batches: A (●), B (●), C (●), and D (●), in processed metabolomics data acquired by analysis of cardiac microtissue intracellular extracts using the polar negative DIMS assay. The separation of samples per exposure batch is observed both* ***a*** *before, and* ***b*** *after, batch correction by robust-spline correction using intra-study QCs (●) as reference (118), indicating inter-batch variation is not of analytical origin. Solid lines show the 95% confidence intervals for each sample group. The dashed line shows the 95% confidence interval around all samples. Data shown is representative of data from all four DIMS assays.*

Statistical analysis employed the availability of batch-specific control samples to negate inter-batch biological variation as a confounding factor in the comparison of responses to xenobiotics between batches.

### **Discovery and annotation of xenobiotic-related features**

As previously (Bowen et al., 2023), three intensity-based filters were applied to the blank-filtered peak-intensity matrices for each of the twelve xenobiotics separately. These filters were (1) retain only features in >50% samples exposed to the high concentration of the given xenobiotic, (2) retain only features in <50% batch-matched control samples, and (3) where features were measured in both exposed and control samples, retain only those where the median intensity in samples exposed to the high concentration of the given xenobiotic is >10x median intensity in batch-matched control samples. Filters were applied to data across all three exposure durations simultaneously. Putative xenobiotic-related features were annotated as described previously, using BEAMSpy and library of SyGMa-predicted biotransformation products for each of the twelve xenobiotics (Bowen et al., 2023).

### **Quantitation of exposure xenobiotics**

***Supplementary Table 5.3 The concentrations of the eight quantitation calibration standards and three quantitation quality controls (QCs) for each xenobiotic.*** *The calibration samples were prepared per batch such that a given sample comprised the relevant concentration of three xenobiotics. Two aliquots of each calibration sample and QC were prepared for analysis.*

| Batch | Xenobiotic | [Quantitation calibration standards] (μM) | | | | | | | | [Quantitation QCs] (μM) | | |
| --- | --- | --- | --- | --- | --- | --- | --- | --- | --- | --- | --- | --- |
|  |  | 1 | 2 | 3 | 4 | 5 | 6 | 7 | 8 | Low | Mid | High |
| A | Acyclovir | 2 | 4 | 8 | 16 | 32 | 64 | 128 | 256 | 6 | 24 | 204.8 |
| A | Clozapine | 0.125 | 0.25 | 0.5 | 1 | 2 | 4 | 8 | 16 | 0.0375 | 1.5 | 12.8 |
| A | Sorafenib | 0.0625 | 0.125 | 0.25 | 0.5 | 1 | 2 | 4 | 8 | 0.1875 | 0.75 | 6.4 |
| B | Buspirone | 0.008 | 0.016 | 0.0313 | 0.0625 | 0.125 | 0.25 | 0.5 | 1 | 0.0234 | 0.0938 | 0.8 |
| B | Doxorubicin | 0.008 | 0.016 | 0.0313 | 0.0625 | 0.125 | 0.25 | 0.5 | 1 | 0.0234 | 0.0938 | 0.8 |
| B | Lapatinib | 0.125 | 0.25 | 0.5 | 1 | 2 | 4 | 8 | 16 | 0.375 | 1.5 | 12.8 |
| C | Erlotinib | 1 | 2 | 4 | 8 | 16 | 32 | 64 | 128 | 3 | 12 | 102.4 |
| C | Idarubicin | 0.0080 | 0.0160 | 0.0313 | 0.0625 | 0.125 | 0.25 | 0.5 | 1 | 0.0234 | 0.0938 | 0.8 |
| C | Sunitinib | 0.0625 | 0.125 | 0.25 | 0.5 | 1 | 2 | 4 | 8 | 0.1875 | 0.75 | 6.4 |
| D | Dasatinib | 0.25 | 0.5 | 1 | 2 | 4 | 8 | 16 | 32 | 0.75 | 3 | 25.6 |
| D | Mebendazole | 2 | 4 | 8 | 16 | 32 | 64 | 128 | 256 | 6 | 24 | 204.8 |
| D | Fluorouracil | 0.5 | 1 | 2 | 4 | 8 | 16 | 32 | 64 | 1.5 | 6 | 51.2 |

### **UHPLC-MS data processing**

Raw data were processed using XCMS in an R environment, with the following parameters: min. peak width: 3; max. peak width: 30; ppm: 10; mzdiff: 0.001; bw: 1; binSize: 0.005; min. frac.: 0.5 per sample group, where sample groups were defined according to batch and exposure conditions. OBI-warp retention time (RT) alignment was also applied with following parameters: response: 10; gapInit: 0.6; gapExtend: 2.4.

Results of preliminary processing revealed substantial shifts in retention time (RT) in the HILIC positive data which was not corrected by OBI-warp RT alignment (Supplementary Fig. 4a). To correct this effect, and maximise accurate alignment of features between all samples, data were split into three groups of samples after peak deconvolution (Supplementary Fig. 4b). OBI-warp RT alignment was applied to each group separately. Functions were subsequently calculated by fitting smooth spline to RT error (difference in RT of reference features between reference sample from group two/three and the group one reference sample) against RT (Supplementary Fig. 5). Reference features were *m/*z-RT features with single peak in both reference samples after XCMS peak density-based feature correspondence using bw: 10 and binSize: 0.005. The calculated functions were used to align RTs of group two and three to those of group one, correcting the observed RT shifts. Data from the three aligned sample groups were merged into a single XCMSSet object before XCMS feature correspondence, generating the aligned peak-intensity matrix.


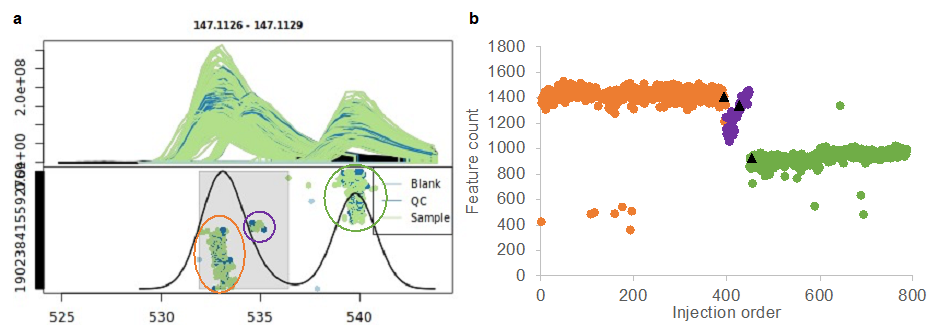
***Supplementary Fig. 4 Retention time shifts observed in HILIC positive dataset.*** ***a*** *Top, extracted ion chromatograms from each sample and, bottom, scatter plot of injection order against retention time, demonstrating the shifts in retention time for one representative m/z-RT feature. Ellipses highlight the three clusters of samples separated based on retention time: group 1 (orange), group 2 (purple) and group 3 (green).* ***b*** *Scatter plot showing relationship between number of features per sample after preliminary XCMS peak picking and correspondence and the injection order, evidencing three sample ‘groups’: group 1 (orange), group 2 (purple) and group 3 (green). The intra-study QCs used as reference samples in the calculation of functions to align retention times between the groups are displayed as black triangles (▲).*


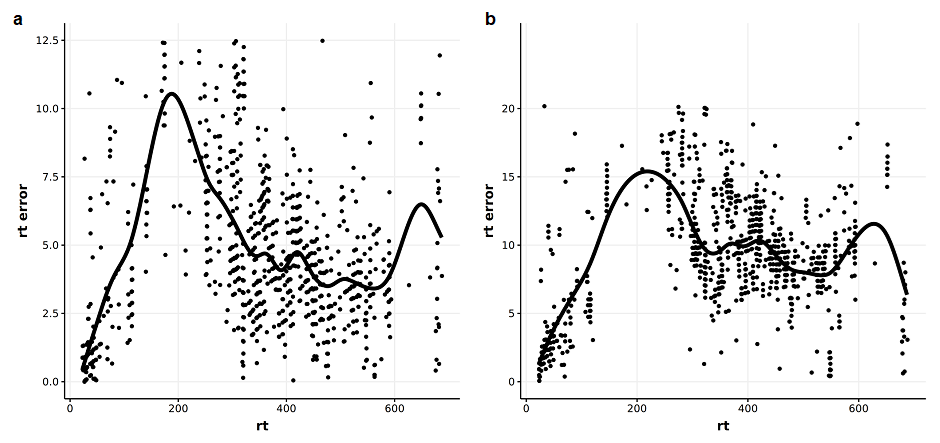


***Supplementary Fig. 5 Smooth spline functions for retention time alignment of HILIC positive data.*** *Plots show the smooth spline functions fitting retention time error of* ***a*** *‘group 2’ samples (retention time in group 2 reference sample minus retention time in group 1 reference sample) and* ***b*** *‘group 3’ samples (retention time in group 3 reference sample minus retention time in group 1 reference sample) against retention time in group 1 reference sample. Sample groups were defined based on visual inspection of peak count after XCMS with no retention time alignment as a function of injection order (Supplementary Fig. 3b).*

### **Annotation of endogenous metabolites measured by UHPLC-MS(/MS)**

Retention time matching to our in-house library was performed using the following criteria: measured *m/z* and RT within ±2.5 ppm and ±10-seconds of library reference values.

UHPLC-MS/MS data were processed using msPurity (Lawson et al., 2017). Parameters were as follows: precursor ion purity >0.5; SNR of fragment ions >3; average across all samples, keeping only fragment ions in at least 50% contributing spectra, to generate a database of MS/MS spectra per assay. Annotations were assigned by comparison to the in-house spectral database, using ±5 ppm mass tolerance for matching of both precursor and product ions. Outputs were filtered to retain only annotations with dot product score >0.8 then aligned to *m/*z-RT features of the MS^1^ peak-intensity matrices using tolerances of ±2.5 ppm and ±20-seconds for *m/z* and RT, respectively.

Outputs of spectral matching against mzCloud database (Compound Discoverer v3.3, Thermo Scientific) were filtered to retain only annotations where HighChem HighRes match score >60. Annotations were aligned to *m/*z-RT features of the MS^1^ peak-intensity matrices using tolerances of ±2.5 ppm and ±20-seconds for *m/z* and RT, respectively.

Aligned annotations for each *m/z*-RT feature were all considered in defining a final compound annotation, with order of preference as follows: (1) MS/MS spectral and RT match to in-house databases of toxicologically relevant metabolites, (2) RT match only to in-house database of toxicologically relevant metabolites, and (3) mzCloud spectral match. Where available data could not distinguish between isomers, multiple compound annotations were collapsed into a single record per *m/z*-RT feature.

##

## **Supplementary Results**


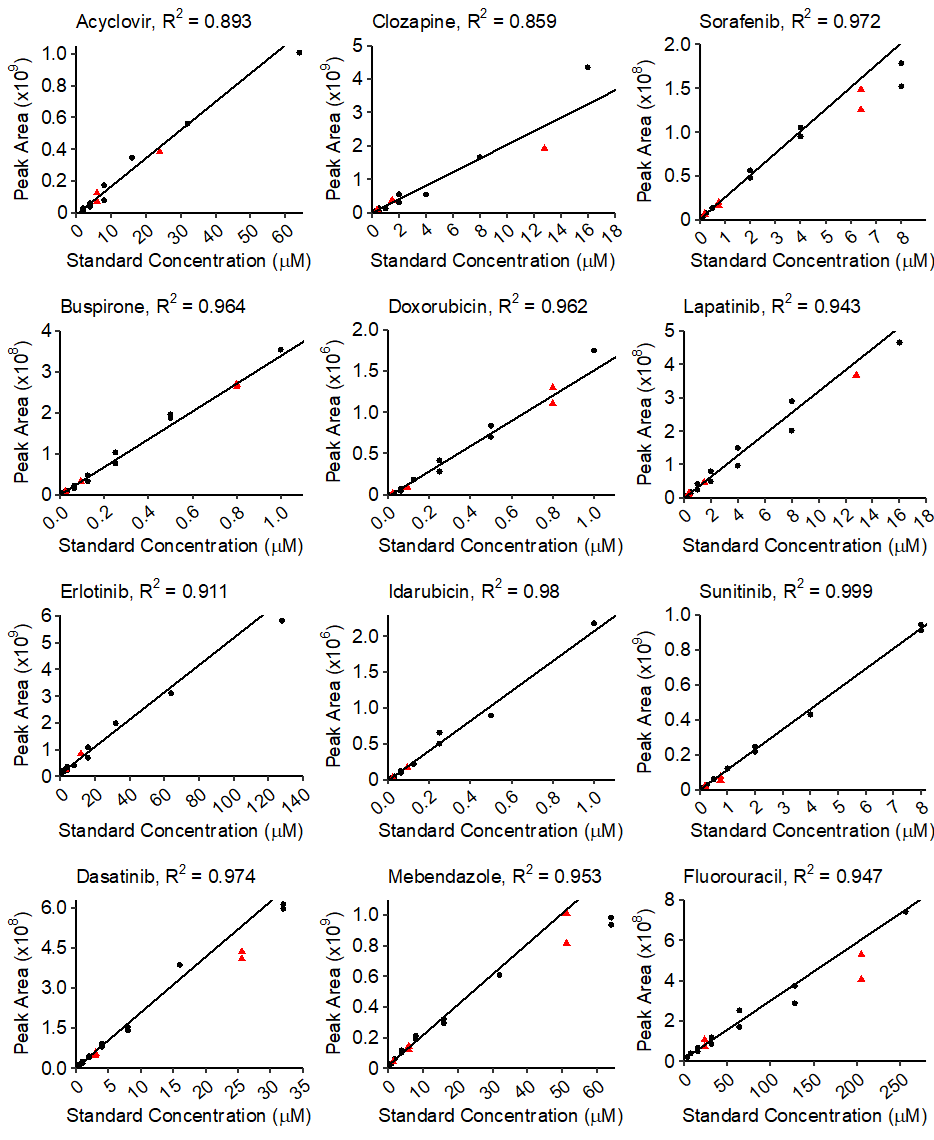


***Supplementary Fig 6 External calibration curves for quantitation of twelve parent xenobiotics.*** *Plots show the integrated area of MS^1^ chromatographic peaks (MSI level 1 identifications based on matching of m/z, retention time and MS/MS to reference standards, Supplementary Fig. 6-7) against the nominal spiked concentration of duplicate quantitation calibration standards (•) for twelve parent xenobiotics. Also shown, are the measurements for duplicate quantitation quality controls (▴). Solid lines represent the calibration function calculated by least squares linear regression analysis with 1/x^2^ weighting. The R^2^, describing the goodness-of-fit of the calibration function, are also reported. Excluded data points (calibration standards and quality controls) are not shown. Calibration precision and accuracy was deemed acceptable for all xenobiotics based on calculated amounts in at least three of six quant-QCs being ±40% to theoretical amounts (accuracy) and the RSDs per quant-QC level <40% (precision). The less stringent acceptance criteria for external calibration curves used here compared to the criteria defined for regulatory studies, e.g., according to FDA guidelines which state that accuracy and precision should be ±15% (255) were guided by the inability to correct for analytical variability and drift. It is anticipated that the inclusion use of (isotopically-labelled) internal standards would enable correction against analytical variation and thus improve the accuracy and precision of quantitation. However, this would come with the risk of negatively impacting, e.g., by ion suppression, the simultaneous measurement of endogenous metabolites in study samples.*

***Supplementary Table 4 External calibration metrics.*** *The ionisation mode of data used for calibration and quantitation, the lower and upper limits of quantitation (LLOQ and ULOQ, respectively) for each of the twelve parent xenobiotics, and the calibration function, as calculated by least squares linear regression, are reported. Also shown is the R^2^ value, used to assess the goodness of fit of calibration standard measurements, the accuracy of quantitation, defined as average absolute difference between calculated concentration and nominal concentration of accepted quantitation quality controls, expressed as a percentage, and the precision of quantitation, defined as the average coefficient of variation across three quantitation quality control duplicates.*

| Xenobiotic | Batch | Ionisation mode | LLOQ (μM) | ULOQ (μM) | Calibration function | Goodness of fit (R^2^) | Accuracy (%) | Precision (%) |
| --- | --- | --- | --- | --- | --- | --- | --- | --- |
| Acyclovir | A | Positive | 2 | 64 |  | 0.893 | 78.7-129.2 | 38.8 |
| Clozapine | A | Positive | 0.125 | 16 |  | 0.859 | 66.3-125.1 | 38.2 |
| Sorafenib | A | Positive | 0.063 | 8 |  | 0.972 | 77.8-128.7 | 10.5 |
| Buspirone | B | Positive | 0.008 | 1 |  | 0.964 | 97.0-104.2 | 3.4 |
| Doxorubicin | B | Positive | 0.016 | 1 |  | 0.962 | 79.3-112.9 | 47.3 |
| Lapatinib | B | Positive | 0.125 | 16 |  | 0.943 | 78.9-126.3 | 36.2 |
| Erlotinib | C | Positive | 1 | 128 |  | 0.911 | 111.7-124.5 | 13.0 |
| Idarubicin | C | Positive | 0.008 | 1 |  | 0.980 | 79.0-94.5 | 19.7 |
| Sunitinib | C | Positive | 0.063 | 8 |  | 0.999 | 63.4-98.8 | 21.1 |
| Dasatinib | D | Positive | 0.25 | 32 |  | 0.974 | 72.8-88.8 | 9.1 |
| Mebendazole | D | Positive | 0.5 | 64 |  | 0.953 | 78.5-108.4 | 13.9 |
| Fluorouracil | D | Negative | 4 | 256 |  | 0.947 | 66.6-137.7 | 22.3 |


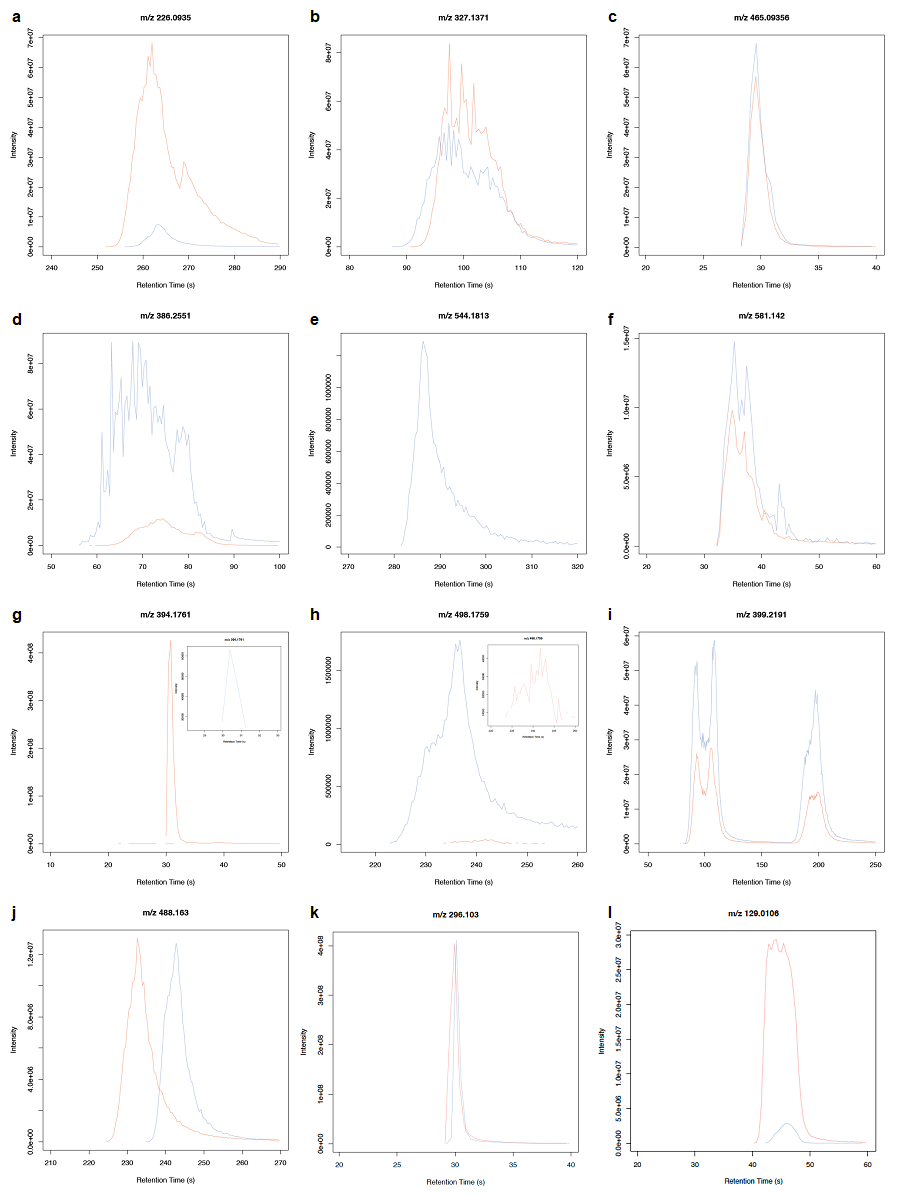
***Supplementary Fig. 7 Extracted ion chromatograms of parent xenobiotic peaks.*** *Extracted ion chromatograms of the [M+H]^+^ ion form of* ***a*** *acyclovir,* ***b*** *clozapine,* ***c*** *sorafenib,* ***d*** *buspirone,* ***e*** *doxorubicin,* ***f*** *lapatinib,* ***g*** *erlotinib,* ***h*** *idarubicin,* ***i*** *sunitinib,* ***j*** *dasatinib, and* ***k*** *mebendazole, as measured by HILIC positive UHPLC-MS, and the [M-H]^-^ ion form of* ***l*** *fluorouracil, as measured by HILIC negative UHPLC-MS, of representative biological sample (red) and preparation of authentic chemical standards (blue). Inset plots (****g*** *and* ***h****), show plot zoomed in on shaded regions.*


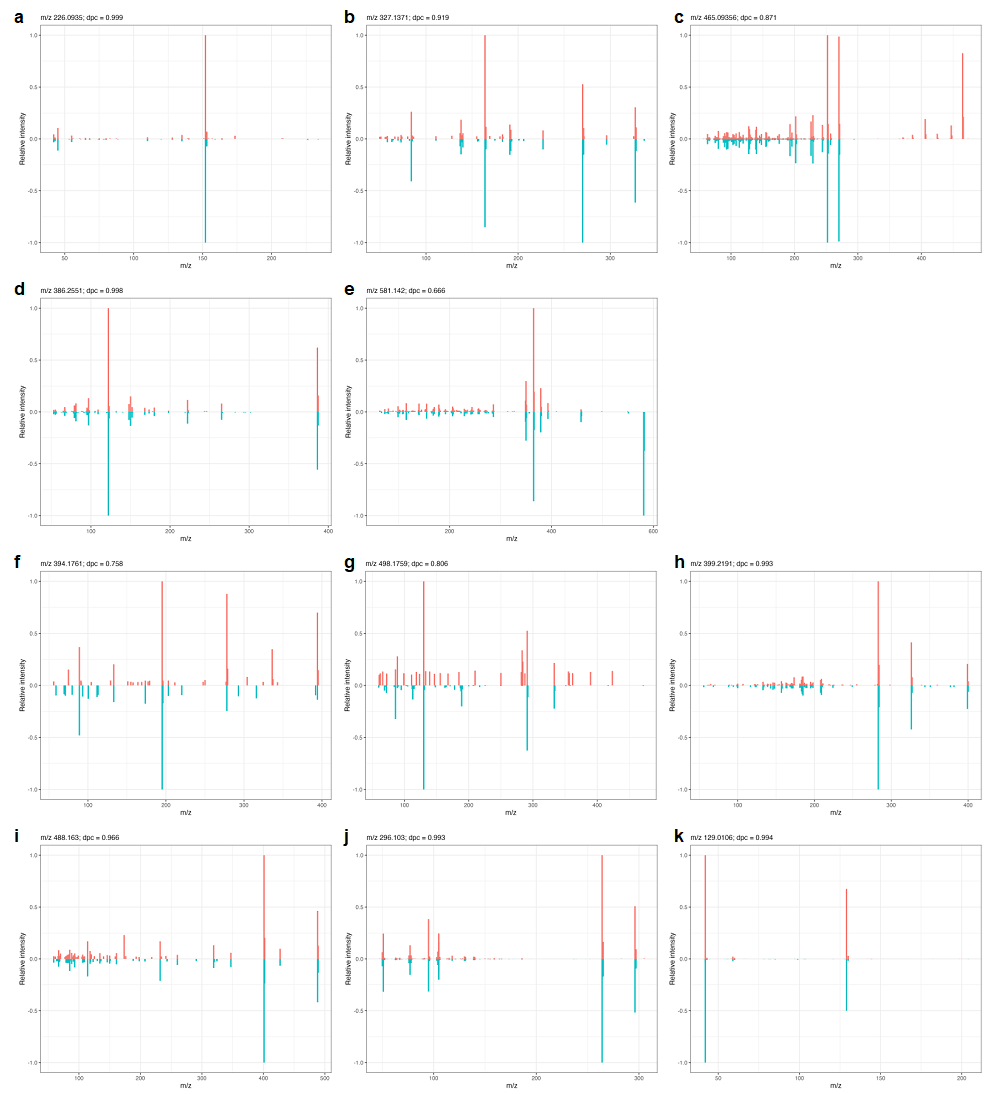
***Supplementary Fig. 8 MS/MS spectra of parent xenobiotics.*** *MS/MS spectra measured by higher-collision energy dissociation-based fragmentation of the [M+H]^+^ ion form (HILIC positive UHPLC-MS/MS ) of* ***a*** *acyclovir,* ***b*** *clozapine,* ***c*** *sorafenib,* ***d*** *buspirone,* ***e*** *lapatinib,* ***f*** *erlotinib,* ***g*** *idarubicin,* ***h*** *sunitinib,* ***i*** *dasatinib and* ***j*** *mebendazole, and of the [M-H]^-^ ion form (HILIC negative UHPLC-MS/MS ) of* ***k*** *fluorouracil. Mirror plots show spectra measured by analysis of representative biological samples (top, red) and authentic chemical standards (bottom, blue). Dot product cosine (dpc) scores, reported in the title of each plot, were >0.6 in all cases, indicating a good match between spectra.*


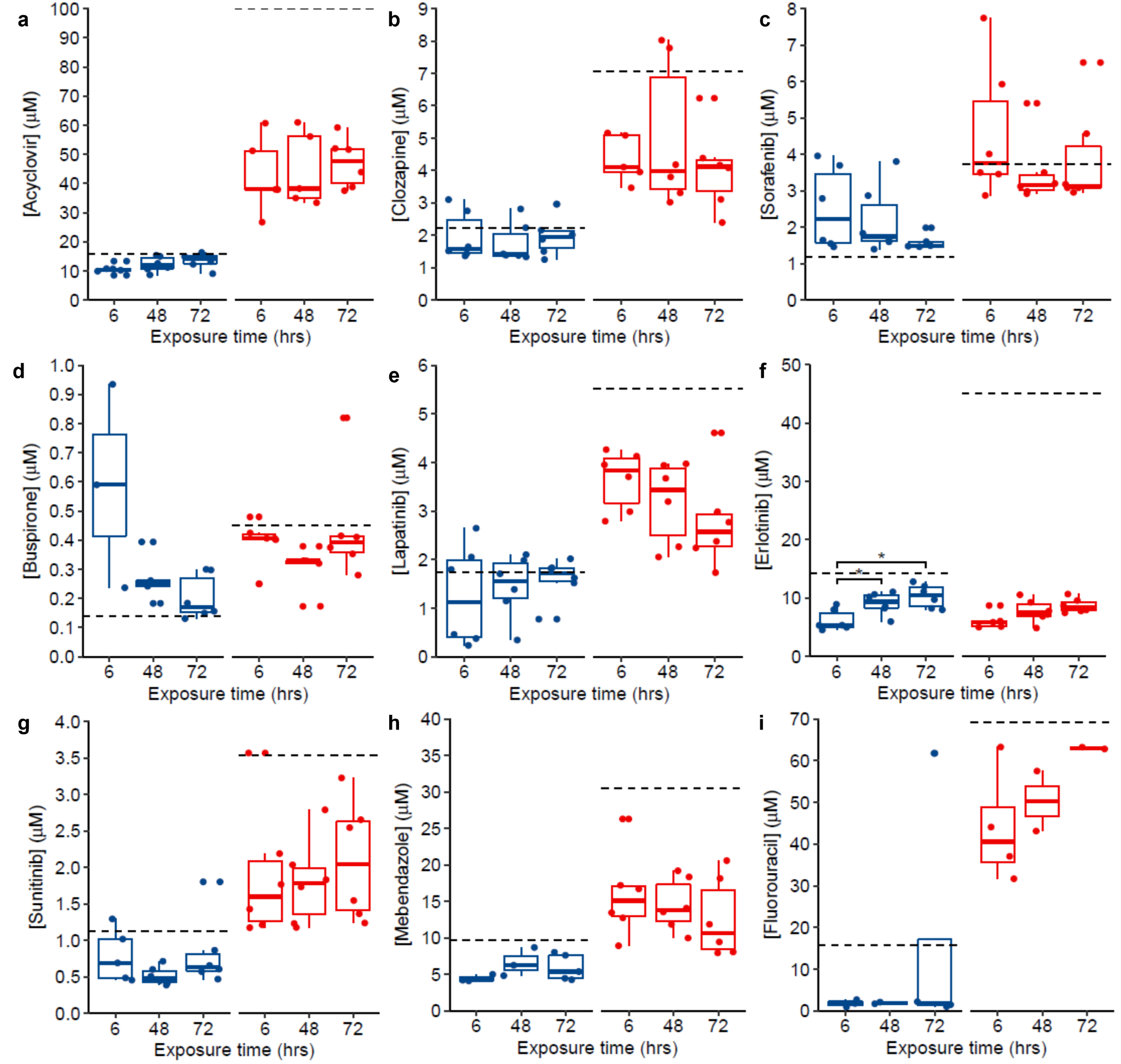


***Supplementary Fig. 9 Quantitation of eleven parent xenobiotics in spent culture medium of cardiac microtissues.*** *Boxplots displaying the concentrations of* ***a*** *acyclovir,* ***b*** *clozapine,* ***c*** *sorafenib,* ***d*** *buspirone,* ***e*** *lapatinib,* ***f*** *erlotinib,* ***g*** *sunitinib,* ***h*** *mebendazole, and* ***i*** *fluorouracil, in samples of pooled spent culture medium from 28 cardiac microtissues, incubated for 6-, 48- and 72-hours (hrs) following addition of the xenobiotics at the nominal concentrations. Concentrations were calculated by external calibration. Blue and red represent calculated concentrations in samples spiked with nominal low and high concentration, respectively. The dashed lines represent the nominal concentrations. Individual points show data from N=6 replicates. * represent p<0.05 significant difference between measured concentrations at each time point (two-sided t-test with Holm’s multiple test correction).*


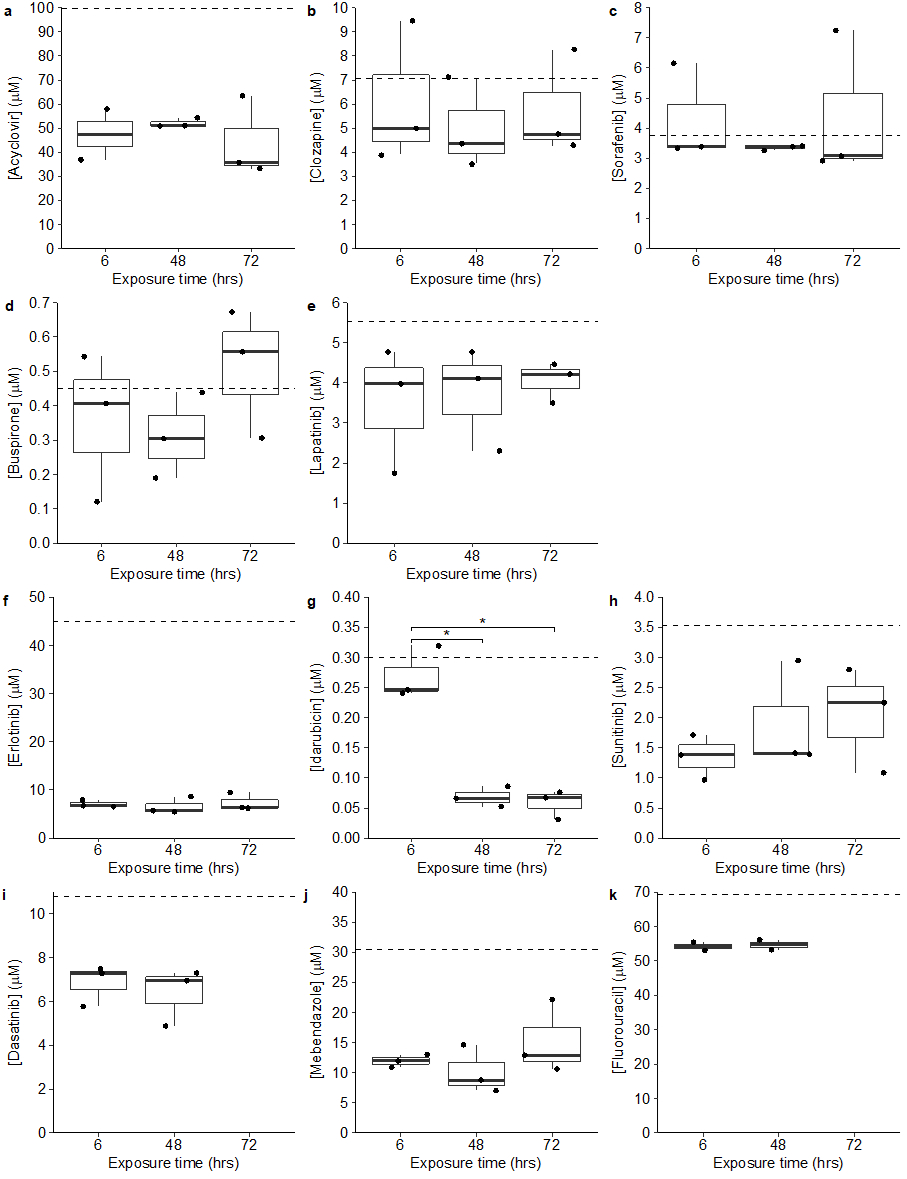
***Supplementary Fig. 10 Quantitation of eleven parent xenobiotics in stability control samples.*** *Boxplots displaying the concentrations of the* ***a*** *acyclovir,* ***b*** *clozapine,* ***c*** *sorafenib,* ***d*** *buspirone,* ***e*** *lapatinib,* ***f*** *erlotinib,* ***g*** *idarubicin,* ***h*** *sunitinib,* ***i*** *dasatinib,* ***j*** *mebendazole, and* ***k*** *fluorouracil in samples composed of culture medium, absent of cardiac microtissues, incubated for 6-, 48- and 72-hours following addition of the xenobiotics at the nominal high concentrations. Concentrations were calculated by external calibration. The dashed lines represent the nominal concentrations. Individual points show data from N = 3 replicates. * p <0.05 significant difference between me*a*sured concentrations at each time point (two-sided t-test with Holm multiple test correction).*

***Supplementary Table 5 Difference between nominal and measured concentrations of eleven quantifiable xenobiotics.*** *The mean calculated concentration of each xenobiotic in spent culture medium of exposed microtissues is reported as a percentage of the nominal concentration. The significance of differences (p-value) was calculated by two-tailed t-test. N = 6 for nominal low and high concentrations, N = 3 for stability controls.*

| Xenobiotic | Low concentration | | High concentration | | Stability controls | |
| --- | --- | --- | --- | --- | --- | --- |
|  | Mean % nominal | *p*-value | Mean % nominal | *p*-value | Mean % nominal | *p*-value |
| Acyclovir | 76.3 | <0.001 | 45.1 | <0.001 | 48.0 | <0.001 |
| Clozapine | 85.1 | 0.038 | 63.6 | <0.001 | 79.8 | 0.077 |
| Sorafenib | 180.8 | 0.001 | 107.0 | 0.455 | 107.5 | 0.605 |
| Buspirone | 220.2 | 0.012 | 85.7 | 0.065 | 87.5 | 0.376 |
| Lapatinib | 81.9 | 0.078 | 57.9 | <0.001 | 68.0 | 0.001 |
| Erlotinib | 60.1 | <0.001 | 16.9 | <0.001 | 15.6 | <0.001 |
| Idarubicin | 55.3 | <0.001 | 34.4 | <0.001 | 44.0 | 0.001 |
| Sunitinib | 62.9 | <0.001 | 54.6 | <0.001 | 50.2 | <0.001 |
| Dasatinib | 64.7 | <0.001 | 54.4 | <0.001 | 61.3 | <0.001 |
| Mebendazole | 59.2 | <0.001 | 30.5 | <0.001 | 40.8 | <0.001 |
| Fluorouracil | 53.1 | 0.299 | 72.7 | 0.004 | 78.7 | <0.001 |

***Supplementary Table 6 Biotransformation products of test xenobiotics discovered in the spent culture media of exposed cardiac microtissues.*** *Biotransformation products discovered by application of the untargeted TK/ADME workflow to datasets acquired by HILIC positive and HILIC negative UHPLC-MS/MS analysis of the spent culture media from exposed cardiac microtissue cultures are listed. Details reported include the parent xenobiotic, an identifier for each biotransformation product, and its molecular formula. Also reported is the ion form by which the biotransformation product was detected, and its measured m/z and chromatographic retention time (RT). The confidence in annotation of each biotransformation product according to MSI and Schymanski confidence levels are reported, and where MS/MS data were available, the m/z and putative molecular formula of MS/MS fragments annotated by MetFrag are listed.*

| Parent xenobiotic | ID | Molecular Formula | Ion form | *m/z* | Rt (min) | MS\| Confidence Level | Schymanski Confidence Level | Annotated MS/MS fragment ions |
| --- | --- | --- | --- | --- | --- | --- | --- | --- |
| Acyclovir | Acy-M1 | C_7_H_9_N_5_O_2_ | [M+H]^+^ | 196.0829 | 1.70 | 3 | 4 | - |
| Acyclovir | Acy-M2 | C_10_H_13_N_5_O_4_ | [M+H]^+^ | 268.1040 | 1.22 | 3 | 4 | - |
| Dasatinib | Das-M6 | C_16_H_15_ClN_6_OS | [M+H]^+^ | 375.0791 | 0.57 | 3 | 4 | - |
| Lapatinib | Lap-M1 | C_22_H_21_ClN_4_O_4_S | [M+H]^+^ | 473.1046 | 1.15 | 3 | 4 | - |
| Lapatinib | Lap-M2 | C_26_H_19_ClFN_3_O_3_ | [M+H]^+^ | 476.1175 | 0.50 | 3 | 4 | - |
| Mebendazole | Meb-M1 | C_16_H_15_N_3_O_3_ | [M+H]^+^ | 298.1186 | 0.52 | 2 | 3 | 95.0491  ([C_6_H_6_O]^+^H^+^)  105.0446 ([C_6_H_4_N_2_]^+^H^+^)  266.0925 ([C_15_H_12_N_3_O_2_]^+^) |
| Sunitinib | Sun-M1 | C_20_H_23_FN_4_O_2_ | [M+H]^+^ | 371.1879 | 4.20 | 2 | 2b | 283.0878 ([C_16_H_13_FN_2_O_2_-H]^+^)  326.1301  ([ C_18_H_17_FN_3_O_2_]^+^) |
| Sunitinib | Sun-M1 | C_20_H_23_FN_4_O_2_ | [M-H]^-^ | 369.1730 | 3.85 | 2 | 2b | 255.0941 ([C_15_H_12_FN_2_O]^-^) |
| Sunitinib | Sun-M2 | C_22_H_27_FN_4_O_3_ | [M+H]^+^ | 415.2140 | 4.17 | 2 | 3 | 326.1300 ([C_18_H_17_FN_3_O_2_]^+^) |

***Supplementary Table 8 Quality assessment of batch- and assay-specific metabolomics data.*** *Metrics used to evaluate the sensitivity and reproducibility of each DIMS and UHPLC-MS assay performed to analyse the intracellular extracts or spent culture media of cardiac microtissues, respectively, are reported. Specifically, the feature count, used to evaluate analytical sensitivity, in each processed peak intensity matrix (one per exposure batch, per assay) is reported. The feature counts are consistent with or exceeded those of previous comparable datasets (Bowen et al., 2021), indicating good analytical sensitivity. The mRSD of normalised feature intensities measured in intra-study QCs is reported as a measure of technical reproducibility. The observed technical variation was consistent with previous studies (Bowen et al., 2021). The range of mRSDs calculated from the normalised feature intensities measured in biological samples belonging to a given exposure condition (total: 21 per batch) are also reported, as an indication of the total metabolic variation between biological replicates. No clear trend with respect to assay, batch, or exposure condition were observed, except values were notably lower for data acquired by HILIC positive UHPLC-MS.*

| Analytical platform | Assay | Batch | Feature Count | Intra-study QC mRSD (%) | Sample group mRSDs (%) |
| --- | --- | --- | --- | --- | --- |
| DIMS | Polar positive | A | 1773 | 20.6 | 23.3 – 42.6 |
|  |  | B | 2003 | 18.6 | 23.5 – 32.6 |
|  |  | C | 1703 | 20.5 | 15.8 – 40.5 |
|  |  | D | 1287 | 20.5 | 21.9 – 37.4 |
|  | Polar negative | A | 4891 | 12.2 | 23.8 – 42.0 |
|  |  | B | 4577 | 12.1 | 17.1 – 34.8 |
|  |  | C | 4150 | 14.5 | 17.1 – 35.0 |
|  |  | D | 4392 | 11.5 | 16.2 – 36.5 |
|  | Lipids positive | A | 4235 | 11.8 | 23.2 – 39.5 |
|  |  | B | 2892 | 13.9 | 21.9 – 33.6 |
|  |  | C | 3414 | 14.2 | 18.5 – 29.5 |
|  |  | D | 2621 | 14.3 | 21.8 – 28.2 |
|  | Lipids negative | A | 4633 | 16.4 | 33.4 – 44.7 |
|  |  | B | 4702 | 12.1 | 20.8 – 41.2 |
|  |  | C | 3524 | 15.4 | 24.4 – 43.2 |
|  |  | D | 5090 | 11.9 | 20.8 – 33.7 |
| UHPLC-MS | HILIC positive | A | 1393 | 17.7 | 14.3 – 19.5 |
|  |  | B | 1519 | 18.1 | 13.3 – 22.0 |
|  |  | C | 1135 | 17.1 | 13.1 – 25.1 |
|  |  | D | 1301 | 15.4 | 16.2 – 22.7 |
|  | HILIC negative | A | 2052 | 14.2 | 18.5 – 30.1 |
|  |  | B | 1857 | 17.5 | 20.4 – 34.0 |
|  |  | C | 1825 | 18.8 | 26.9 – 54.8 |
|  |  | D | 1951 | 17.0 | 21.4 – 34.4 |


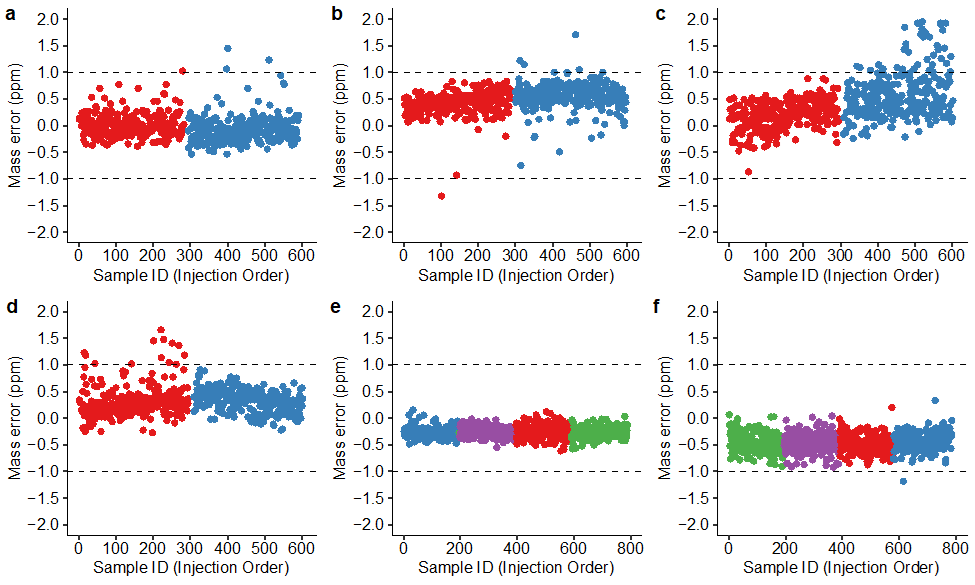


***Supplementary Fig. 11 Mass error of internal standard measurements across analytical sequences.*** *The error, in parts per million (ppm), of the measured m/z compared to the theoretical m/z of the,* ***a*** *and* ***e*** *[M+H]^+^, and,* ***b*** *and* ***f*** *[M-H]^-^, ion forms of L-tryptophan-d5-indole, and,* ***c*** *[M+H]^+^, and,* ***d*** *[M+acetate]^-^, ion forms of dodecylphosphorylcholine-d38. Measurements were made by* ***a*** *DIMS polar positive,* ***b*** *DIMS polar negative,* ***c*** *DIMS lipids positive,* ***d*** *DIMS lipids negative,* ***e*** *HILIC positive UHPLC-MS and* ***f*** *HILIC negative UHPLC-MS analytical assays. Colour of points in each panel indicate analytical batch:* ***a-d*** *an analytical batch is composed of two exposure batches (red: analytical batch 1, blue: analytical batch 2),* ***e-f*** *analytical batches are equivalent to exposure batches (blue: B, purple: D, green: C, red: A). Measurements were within ±1 ppm of theoretical m/z throughout analysis, with the exception of a few outliers (0.7%, 1.4%, 8.2%, 2.3%, 0.0%, and 0.1% of samples measured by* ***a*** *DIMS polar positive,* ***b*** *polar negative,* ***c*** *lipids positive,* ***d*** *lipids negative, and* ***e*** *UHPLC-MS HILIC positive,* ***f*** *HILIC negative, respectively). This is deemed excellent mass accuracy, enhancing the potential to gain biological insights by enabling the use of a smaller mass tolerance for metabolite annotation, reducing the proportion of false positive assignments.*


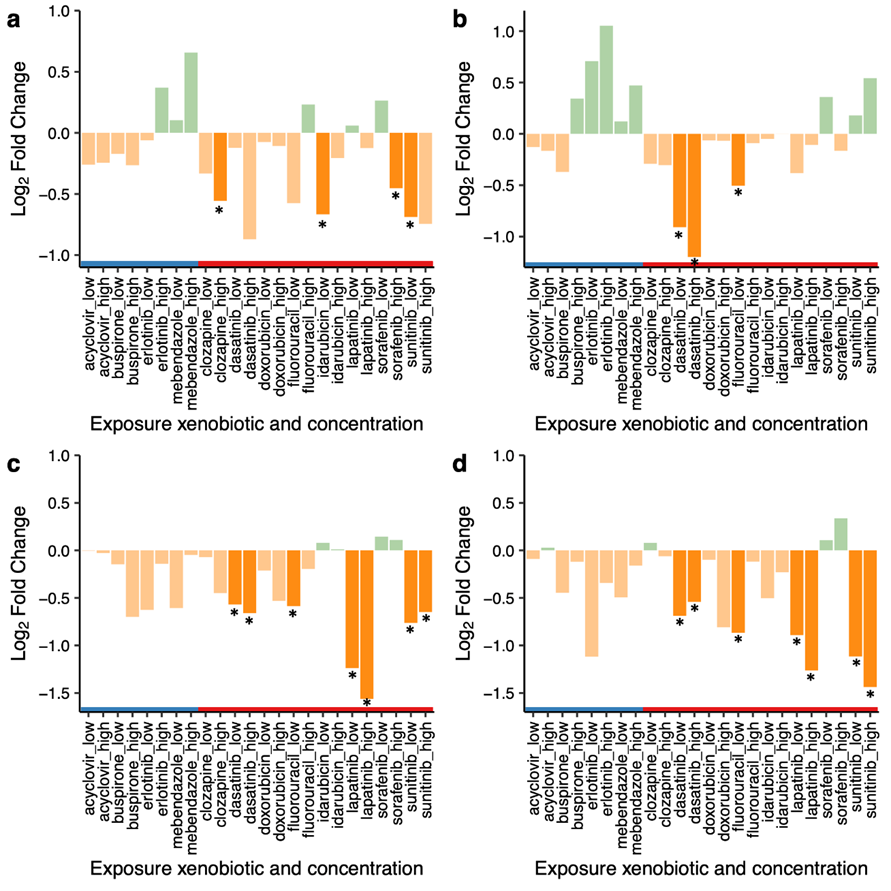


***Supplementary Fig. 12 Structural cardiotoxin-specific significant intracellular metabolic perturbations in cardiac microtissues.*** *Plots show the average log_2_ fold change of features putatively annotated as* ***a-b*** *glycerol-3-phosphoethanolamine,* ***c*** *Cer 33:1;O_2_ and* ***d*** *Cer 43:2;O_2_ in response to twelve xenobiotics, each at two concentrations. Responses were measured after a 48-hour, and b-d 72-hour exposures. The colours on the x-axes indicate the structural cardiotoxicity label of each xenobiotic, blue: not structurally cardiotoxic, red: structurally cardiotoxic. The colour of the bars indicates the direction of change in the relative abundance of features following xenobiotic exposure relative to batch- and time-matched controls, green: increase, orange: decrease. Darker shading and * highlight changes that were found significant according to Welch’s t-test (p <0.05, at least one representative feature).*


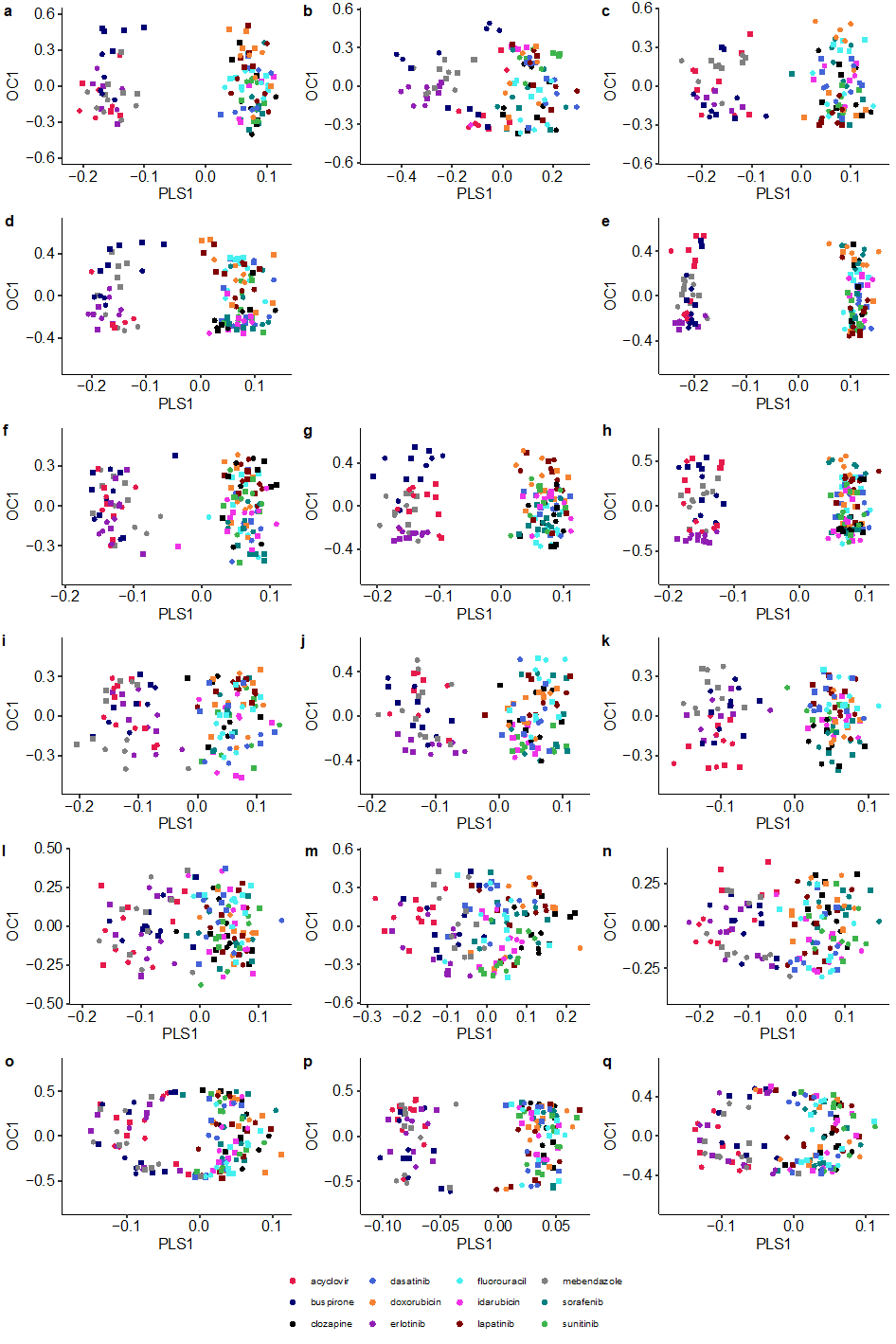
***Supplementary Fig. 13 OPLS-DA models predictive of structural cardiotoxicity.*** *OPLS-DA scores plots showing the predictive and first orthogonal latent variables of a model which discriminates between the responses of cardiac microtissues induced by* ***a****,* ***d****,* ***f,*** ***i****,* ***I****,* ***o*** *6- ,* ***b****,* ***g****,* ***j****,* ***m****,* ***p*** *48-, and* ***c****,* ***e****,* ***h****,* ***k****,* ***n****,* ***q*** *72-hour exposure to structurally cardiotoxic xenobiotics compared with non-structurally cardiotoxic xenobiotics. Models were built from subsets of predictive features, determined according to VIP score (VIP ≥ 1) calculated in preliminary models, measured by* ***a-c*** *polar positive,* ***d-e*** *polar negative,* ***f-h*** *lipids positive, and* ***i-k*** *lipids negative DIMS-based analysis of intracellular extracts, and* ***l-n*** *HILIC positive and* ***o-q*** *HILIC negative UHPLC-MS analysis of spent culture media of cardiac microtissues. Shaded regions represent the 95% confidence intervals for structurally cardiotoxic (red) and non-structurally cardiotoxic (blue) xenobiotics. Responses to both low (■) and high (●) concentrations of the twelve xenobiotics were used for model training. Intensity measurements were corrected for batch and baseline temporal effects by normalisation against the median intensities in batch and time matched DMSO controls prior to analysis.*

***Supplementary Table 11 Performance of OPLS-DA models.*** *The table reports performance metrics for the refined OPLS-DA models, built for each analytical assay and exposure duration combination (18 total) using data matrices of the most predictive features, determined by VIP score (VIP ≥1) calculated in preliminary models. The number of features in the input data and the number of orthogonal components used in each model are shown. Performance metrics reported include model sensitivity, specificity, area under the curve (AUC) and balanced error rate (BER), each calculated by k-fold cross-validation (k = 5), and p-values assessing the significance of the BER as calculated by permutation tests with k-fold cross validation.*

| Platform | Assay | Exposure duration (hours) | Number of features | Number of orthogonal components | Specificity | Sensitivity | AUC | BER | BER  *p*-value |
| --- | --- | --- | --- | --- | --- | --- | --- | --- | --- |
| DIMS | Polar positive | 6 | 412 | 12 | 0.83 | 0.72 | 0.86 | 0.215 | 0.01 |
|  |  | 48 | 351 | 3 | 0.85 | 0.86 | 0.93 | 0.140 | 0.01 |
|  |  | 72 | 307 | 8 | 0.89 | 0.88 | 0.95 | 0.135 | 0.01 |
|  | Polar negative | 6 | 833 | 10 | 0.74 | 0.87 | 0.89 | 0.165 | 0.01 |
|  |  | 48 | 1171 | 13 | 0.80 | 0.96 | 0.95 | 0.103 | 0.01 |
|  |  | 72 | 1154 | 13 | 0.85 | 0.98 | 0.98 | 0.098 | 0.01 |
|  | Lipids positive | 6 | 744 | 10 | 0.67 | 0.87 | 0.85 | 0.284 | 0.01 |
|  |  | 48 | 705 | 9 | 0.86 | 0.94 | 0.97 | 0.096 | 0.01 |
|  |  | 72 | 644 | 11 | 0.84 | 1.00 | 0.99 | 0.091 | 0.01 |
|  | Lipids negative | 6 | 1172 | 7 | 0.60 | 0.66 | 0.63 | 0.408 | 0.06 |
|  |  | 48 | 1074 | 8 | 0.69 | 0.85 | 0.83 | 0.278 | 0.01 |
|  |  | 72 | 1112 | 8 | 0.67 | 0.58 | 0.68 | 0.353 | 0.01 |
| UHPLC-MS | HILIC positive | 6 | 240 | 3 | 0.68 | 0.79 | 0.83 | 0.291 | 0.01 |
|  |  | 48 | 220 | 4 | 0.74 | 0.67 | 0.81 | 0.277 | 0.01 |
|  |  | 72 | 232 | 9 | 0.68 | 0.79 | 0.83 | 0.291 | 0.01 |
|  | HILIC negative | 6 | 378 | 4 | 0.62 | 0.72 | 0.79 | 0.300 | 0.01 |
|  |  | 48 | 419 | 8 | 0.68 | 0.70 | 0.75 | 0.346 | 0.01 |
|  |  | 72 | 361 | 4 | 0.67 | 0.83 | 0.84 | 0.230 | 0.01 |


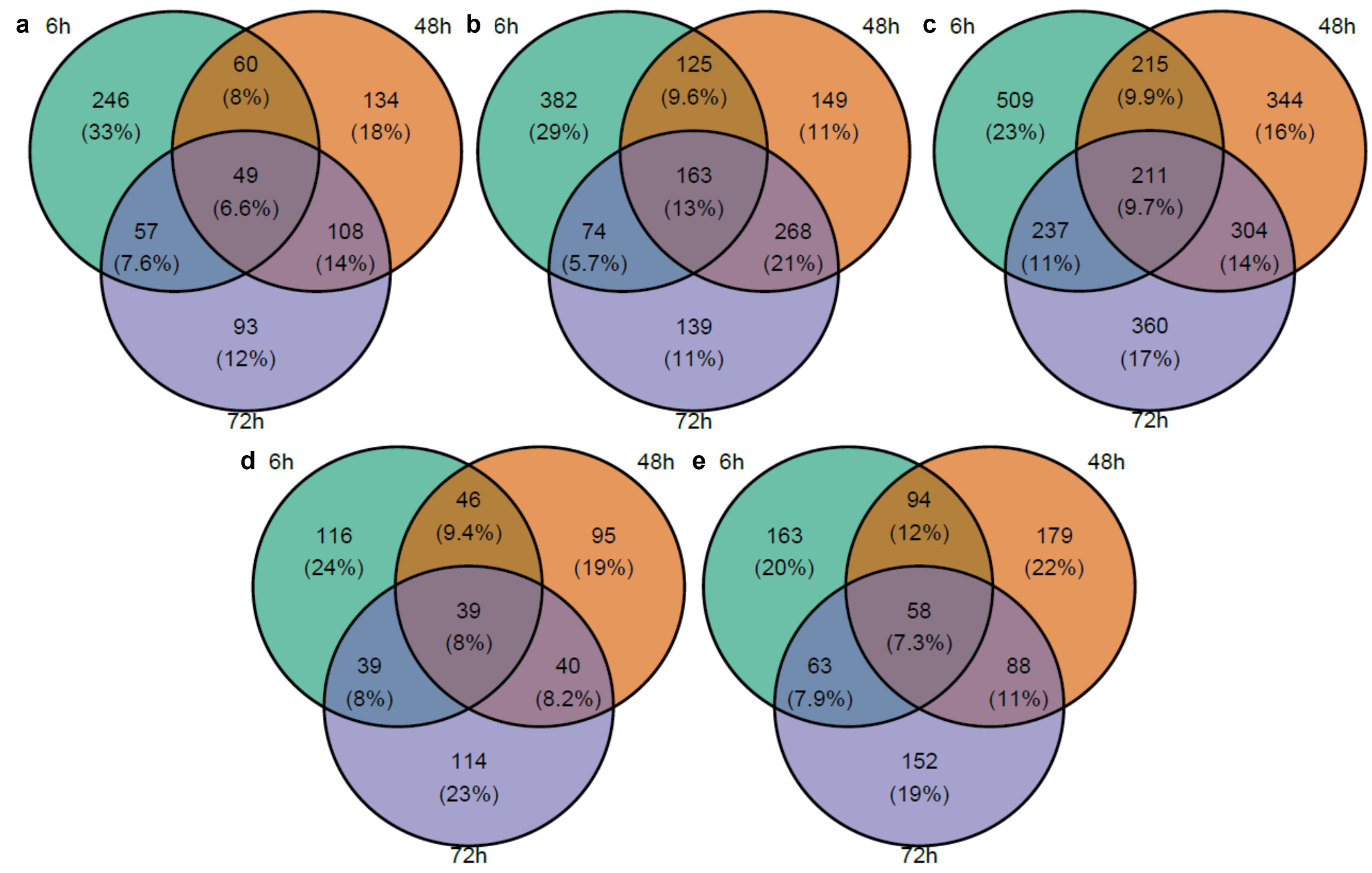


***Supplementary Fig. 14 Conservation of predictive features over time.*** *Venn diagrams comparing the m/z or m/z-RT features found to be predictive of structural cardiotoxicity (VIP >1) in preliminary models built using the entire dataset and used to build the refined OPLS-DA models, across three exposure durations. Data were acquired by* ***a*** *polar positive,* ***b*** *lipids positive,* ***c*** *lipids negative nESI-DIMS analysis of intracellular extracts,* ***d*** *HILIC positive and* ***e*** *HILIC negative UHPLC-MS analysis of spent culture media of cardiac microtissues.*


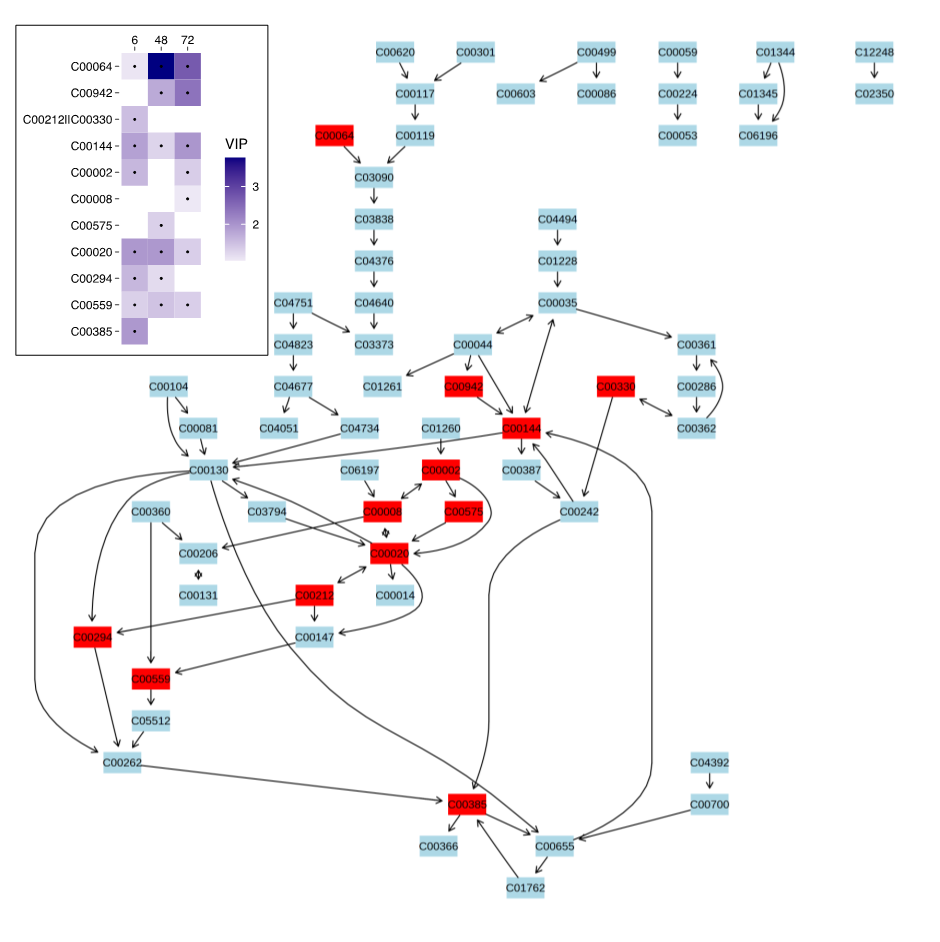


***Supplementary Fig. 15 Intracellular levels of purine metabolism intermediates in cardiac microtissues are predictive of the structural cardiotoxicity potential of exposure xenobiotics.*** *The KEGG purine metabolism pathway (PATHWAY: map00230, Kanehisa, 2000). Intermediates of the pathway found to be predictive of structural cardiotoxicity by OPLS-DA of intracellular metabolomics data from xenobiotic exposed cardiac microtissues are highlighted in red. The inset heatmap (top left) displays the average VIP scores for the metabolites (purine metabolism intermediates) included in the refined OPLS-DA models. The average VIP score shown for each metabolite per exposure duration is the mean VIP score of m/z features within the refined OPLS-DA models that were putatively annotated as the given metabolite. Metabolites are referred to by their KEGG identifiers (C00064: L-glutamine; C00942: cyclic-GMP; C00330: deoxyguanosine; C00144: guanosine monophosphate; C00002: adenosine triphosphate; C00008: adenosine diphosphate; C00575: cyclic-AMP; C00020: adenosine monophosphate; C00212: adenosine; C00294: inosine; C00559: deoxyadenosine; C00385: xanthine).*

**Supplementary References**

Bowen TJ, Hall AR, Lloyd GR, Weber RJM, Wilson A, Pointon A, Viant MR. (2021) An Extensive Metabolomics Workflow to Discover Cardiotoxin-Induced Molecular Perturbations in Microtissues. Metabolites. 11(9):644. doi: 10.3390/metabo11090644.

Bowen TJ, Southam AD, Hall AR, Weber RJM, Lloyd GR, Macdonald R, Wilson A, Pointon A, Viant MR. (2023) Simultaneously discovering the fate and biochemical effects of pharmaceuticals through untargeted metabolomics. Nat Commun. 14(1):4653. doi: 10.1038/s41467-023-40333-7.

Kanehisa M. KEGG: Kyoto Encyclopedia of Genes and Genomes. Nucleic Acids Research. 2000;28(1):27-30.

Kang W-Y, Thompson PT, El-Amouri SS, Fan TWM, Lane AN, Higashi RM. Improved segmented-scan spectral stitching for stable isotope resolved metabolomics (SIRM) by ultra-high-resolution Fourier transform mass spectrometry. Analytica Chimica Acta. 2019;1080:104-15.

Lawson TN, Weber RJ, Jones MR, Chetwynd AJ, Rodri Guez-Blanco G, Di Guida R, et al. msPurity: Automated Evaluation of Precursor Ion Purity for Mass Spectrometry-Based Fragmentation in Metabolomics. Anal Chem. 2017;89(4):2432-9.

Sarvin B, Lagziel S, Sarvin N, Mukha D, Kumar P, Aizenshtein E, et al. Fast and sensitive flow-injection mass spectrometry metabolomics by analyzing sample-specific ion distributions. Nature Communications. 2020;11(1).

Sostare E, Lawson TN, Saunders LR, Colbourne JK, Weber RJM, Sobanski T, et al. (2022) Knowledge-driven approaches to create the MTox700+ metabolite panel for predicting toxicity. Toxicological Sciences.

Sumner LW, Amberg A, Barrett D, Beale MH, Beger R, Daykin CA, Fan TW, Fiehn O, Goodacre R, Griffin JL, Hankemeier T, Hardy N, Harnly J, Higashi R, Kopka J, Lane AN, Lindon JC, Marriott P, Nicholls AW, Reily MD, Thaden JJ, Viant MR. (2007) Proposed minimum reporting standards for chemical analysis Chemical Analysis Working Group (CAWG) Metabolomics Standards Initiative (MSI). Metabolomics. 3(3):211-221. doi: 10.1007/s11306-007-0082-2.
